# New Chiral Phosphoramidite Complexes of Iron as Catalytic Precursors in the Oxidation of Activated Methylene Groups

**DOI:** 10.3390/molecules15042631

**Published:** 2010-04-12

**Authors:** Pushkar Shejwalkar, Nigam P. Rath, Eike B. Bauer

**Affiliations:** 1 University of Missouri - St. Louis, Department of Chemistry and Biochemistry, One University Boulevard, St. Louis, MO 63121, USA; E-Mail: pssgk8@umsl.edu (P.S.); 2 Department of Chemistry and Biochemistry and Center for Nanoscience, University of Missouri - St. Louis, One University Boulevard, St. Louis, MO 63121, USA; E-Mail: rathn@umsl.edu (N.P.R.)

**Keywords:** iron, phosphoramidites, X-ray, chiral at metal, benzylic oxidations

## Abstract

New phosphoramidite complexes of iron were synthesized and structurally characterized. Reaction of the known chiral phosphoramidites (RO)_2_PNR’_2_ (R = binaphthyl, R’ = CH_3_, **1a**; R = binaphthyl, R’ = benzyl, **1b**) with [FeBr(Cp)(CO)_2_] afforded the title compounds [FeBr(Cp)(CO)(**1a**,**b**)] (**4a,b**) in 34 and 65 % isolated yields as mixtures of diastereomers, since both the metal and the ligand are stereogenic. Similarly, reaction of **1b** with [Fe(Cp)I(CO)_2_] in the presence of catalytic [Fe(Cp)(CO)_2_]_2_ afforded [Fe(Cp)I(CO)(**1b**)] (**5b**) in 81% yield as a mixture of diastereomers. The molecular structures of **4a**, **4b** and **5** were determined, revealing a pseudo octahedral coordination geometry about the iron center. The new metal complexes are catalytically active in the oxidation of benzylic methylene groups to the corresponding ketones, utilizing *t*-BuOOH as oxidant (2 mol% catalyst, 36 h, room temperature, 31−80% yield).

## 1. Introduction

The investigation of transition metal catalyzed organic transformations is a research area of high interest in chemistry. Catalysis can improve selectivities, decrease the energy required for reactions, and allows for enantioselection. The mainstream of organometallic catalysis focuses on transition metals such as Pd, Rh, Ir, Ru, or Au. Iron, however, is a cheap, abundant, non-toxic and environmentally friendly alternative to well-established transition metals in catalysis. Consequently, an increasing number of publications are being devoted to the development of iron based catalyst systems [1,2,3,4,5,6,7,8] that have been applied as catalysts in a variety of organic transformations [9,10,11,12,13,14,15,16,17,18,19,20,21], including oxidation reactions [22,23,24,25]. 

Typically, *in situ* catalyst systems are employed, consisting of an iron source and a ligand [22]. The deployment of well-defined, preformed iron complexes is less common, but such complexes allow not only for kinetic investigations but also for determination of the impact of ligand structure on the activity. 

**Figure 1:**
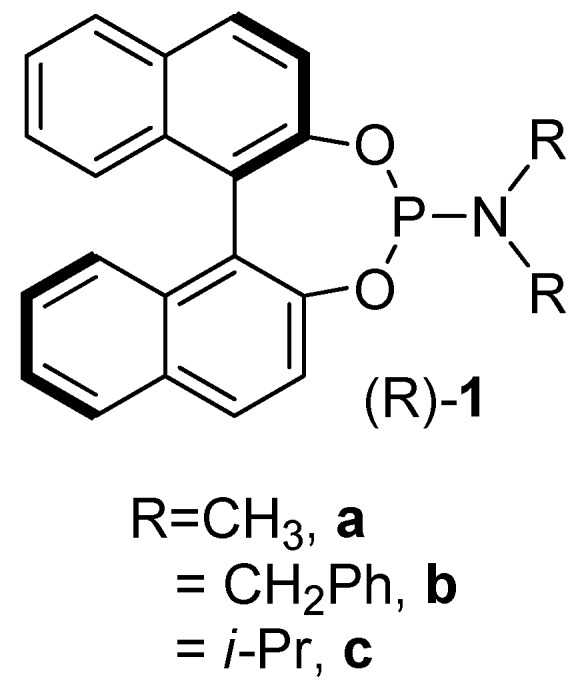
Phosphoramidite ligands.

Phosphoramidites (**1**, Figure 1) are a monodendate ligand class, which have recently gained prominence in transition metal complex catalyzed organic transformations [26,27,28]. Originally utilized by Feringa [28], phosphoramidites have been employed as ligands in catalytically active metal complexes for a variety of organic reactions such as conjugate enone addition reactions [29], hydrogenations [30], allylic substitutions [31], cycloadditions [32], vinylations [33], and other reactions. We have recently reported new ruthenium phosphoramidite complexes and their catalytic activation in the formation of β-oxo esters from propargylic alcohols and carboxylic acids [34] and in the Mukaiyama aldol reaction [35]. As far as we know, phosphoramidite complexes have never been employed in oxidation reactions thus far. 

To the best of our knowledge, iron complexes containing the ligands **1** shown in Figure 1 are unknown thus far. However, iron based organometallic architectures bearing ligands reminiscent of those in Figure 1 have been previously described in the literature. For example, rearrangements in iron phosphoranide complexes led to a phosphoramidite ligand coordinated to iron [36,37]. Nakazawa and Miyosh showed that acyclic diaminooxyphosphine iron complexes undergo Arbuzov-like dealkylation reactions to give amino-substituted oxophosphoranes [38]. Structurally related iron iminophosphorane complexes are known as well [39]. Some of these complexes are not very thermally stable and they have not been applied as catalysts. 

We were interested in synthesizing iron complexes of the commonly employed phosphoramidite ligands **1** shown in Figure 1. We were furthermore interested in applications of these new iron phosphoramidite complexes in catalysis. We are especially attracted by iron catalyzed oxidations of alkanes [40,41]. Alkanes are rather unreactive, and their oxidation increases a structural complexity that allows for further transformations. 

This study describes the synthesis of new iron phosphoramidite complexes of the general formula [FeX(Cp)(CO)(**1**)] (X=Br, I). We furthermore applied the complexes as catalysts in the oxidation of activated benzylic methylene groups with *t*-BuOOH to obtain the corresponding ketones. 

## 2. Results and Discussion

### 2.1. Synthesis of the Iron Phosphoramidite Complexes

First, synthetic access to iron complexes bearing phosphoramidite ligands was targeted. The iron carbonyl complex [FeBr(Cp)(CO)_2_] (**2**) [42] is known to undergo thermal displacement of Br^–^ by a neutral ligand L to give ionic complexes of the general formula [Fe(Cp)(CO)_2_L][Br] [43] or to undergo CO displacement to give neutral complexes of the formula [FeBr(Cp)(CO)L] (L=phosphonite) [36]. Displacement of one of the CO ligands by phosphites or phosphonites is the major reaction when performed under UV radiation [44]. Bromide exchange appears to take place preferably with anionic nucleophiles such as acetylides [45,46] to give neutral iron complexes. In the same way, the iodo complex [Fe(Cp)I(CO)_2_] (**3**) [47] undergoes ligand exchange to either give [Fe(Cp)(CO)_2_L][I] or [Fe(Cp)I(CO)L] (L=PPh_3_) [43,48]. It has been reported that either [Fe(Cp)(CO)_2_]_2_ or Me_3_NO·2H_2_O can catalyze the reaction [49]. 

**Scheme 1:**
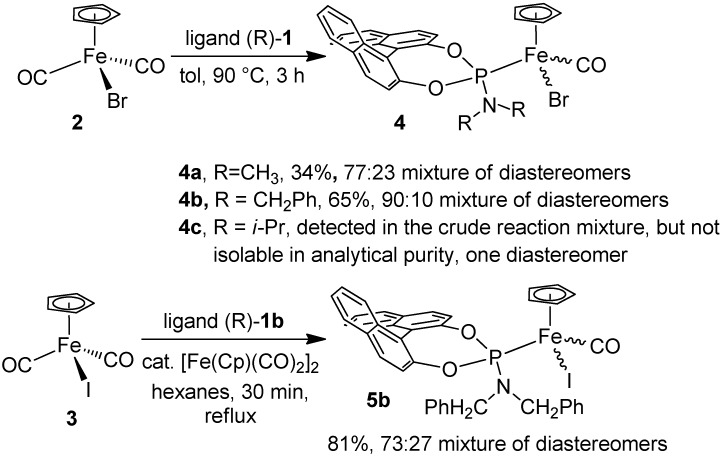
Iron Phosphoramidite Complex Syntheses.

Accordingly, when the complex [FeBr(Cp)(CO)_2_] (**2**) was heated with one equivalent of the phosphoramidite ligand **1a** in toluene for 3 h at 90 °C, the complex [FeBr(Cp)(CO)(**1a**)] (**4a**) was isolated in 34% yield as a greenish solid (Scheme 1). Applying identical conditions with ligand **1b** gave the complex [FeBr(Cp)(CO)(**1b**)] (**4b**) in 65% isolated yield. Significantly, when the *i*-Pr ligand **1c** was employed under these conditions, a clean reaction to the corresponding iron complex was not achieved. The crude reaction mixture contained the corresponding iron complex **4c**, as shown by MS and NMR data. However, workup efforts resulted in **4c** being obtained in only 60% spectroscopic (^1^H-NMR) purity. The isolated material frequently contained the free phosphoramidite ligand **1c**, suggesting ongoing decomposition of **4c** due to ligand loss. Among the ligands **1**, the isopropyl ligand **1c** is the only one bearing a secondary carbon atom on the nitrogen. Thus, **1c** presumably generates too much steric congestion about the iron center, resulting in an unstable complex. 

Similarly, the iodo complex [Fe(Cp)I(CO)_2_] (**3**) was heated with ligand **1b** in the presence of a catalytic amount of [Fe(Cp)(CO)_2_]_2_ (Scheme 1), which gave the neutral complex **5b** in 81% yield. Significantly, when **1a** and **1c** were employed as ligands under similar conditions, incomplete reaction occurred, resulting in an inseparable mixture of compounds. 

The new iron complexes **4a**, **4b** and **5b** were characterized by NMR (^1^H, ^13^C, ^31^P), MS, IR and **4a**,**b** by microanalysis. The coordination of the ligands **1a**,**b** was best observed by a downfield shift of the ^31^P-NMR signals. The free phosphoramidite ligands **1a**,**b** exhibited signals around 150 ppm, whereas the corresponding complexes **4a**,**b** and **5b** showed resonances between 196.3 and 200.7 ppm. The IR spectra showed single absorptions at 1,971 (**4a**, **5b**) and 1,978 cm^–1^ (**4b**), as expected for monocarbonyl complexes. The precursor complex [FeBr(Cp)(CO)_2_] exhibits two ν_C≡O_ absorptions at 1,995 and 2,049 cm^–1^[42]. The CO ligands were also observed in the ^13^C NMR spectra, giving signals between 216.9 and 217.7 ppm (*J*_CP_ = 21.8 to 45.3 Hz). The MS spectra are also in accordance with the proposed structures. For **4a** and **4b**, molecular ion peaks were not observed in the FAB MS spectra, but peaks for the corresponding sodium adducts [4+Na]^+^ appeared. However, their FAB MS spectra complexes exhibited a diagnostic fragmentation pattern resulting from CO and/or Br loss. For complex **5b**, this pattern also was observed, including the molecular ion peak [**5b**]^+^. 

For the metal complex syntheses, enantiopure ligands were employed. The resulting complexes **4a**,**b** and **5b** are stereogenic at the metal and at the ligands, and accordingly, two diastereomers are formed during synthesis, as seen in the NMR spectra. The complexes **4a**,**b** and **5b** were thus obtained as a 77:23, 90:10 and 73:27 mixture of diastereomers, respectively. Some signals in the ^1^H, ^13^C and ^31^P NMR spectra are doubled, and the diastereomeric ratios were determined by ^1^H- and ^31^P-NMR. All efforts to isolate one of the complexes in diastereomerically pure form have failed to date, but it was possible to enrich the major diastereomer for complex **4a** to a 85:15 diastereomeric ratio. Complex **4c** could not be isolated analytically pure, but its crude ^1^H- and ^31^P-NMR spectra showed only one diastereomer, which would be in accordance with the larger steric bulk of the phosphoramidite ligand **1c**. 

**Figure 2:**
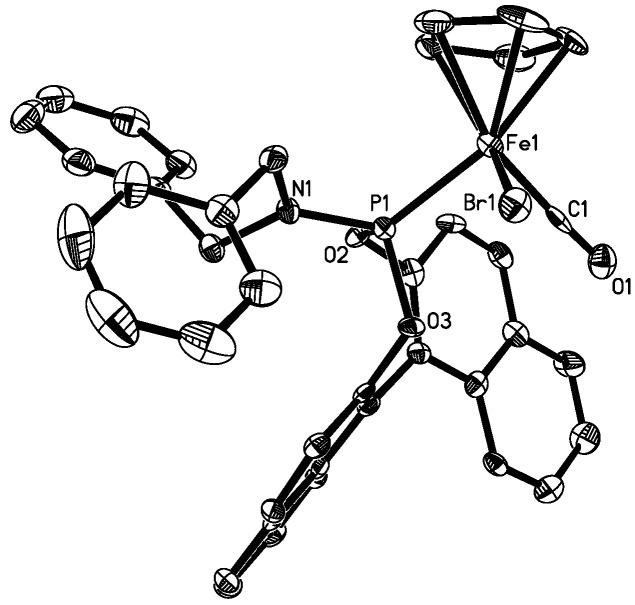
Molecular structure of one of the diastereomers of **4b** (depicted with 65% probability ellipsoids, H atoms, and solvents are omitted for clarity). Key bond lengths and bond angles are listed in Table 2.

**Figure 3:**
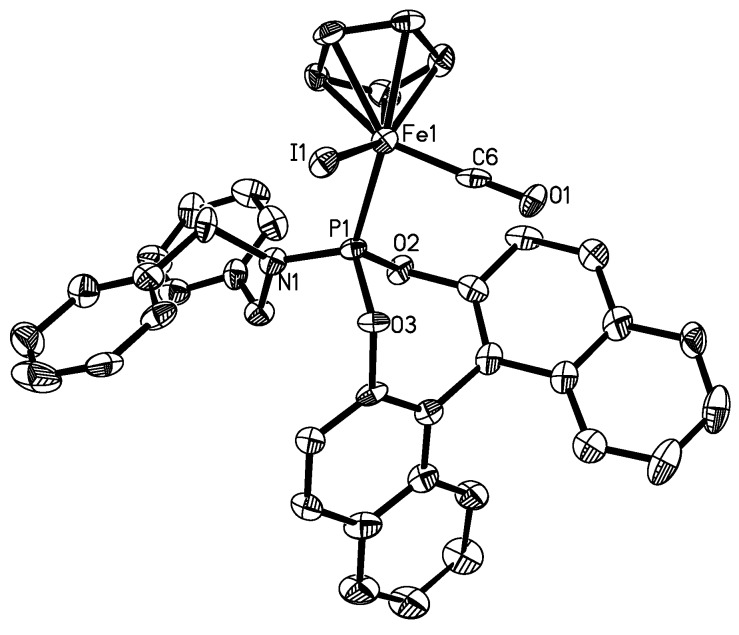
Molecular structure of one of the diastereomers of **5b** (depicted with 65% probability ellipsoids, H atoms, and solvents are omitted for clarity). Key bond lengths and bond angles are listed in Table 2.

Although separation of the diastereomers in bulk has failed to date, we were able to obtain crystals of X-ray quality in the recrystallized material of the complexes **4b** and **5b**, and the X-ray structures were determined (Table 1 and Experimental section). The molecular structures are shown in Figure 2 and Figure 3, and key structural data is given in Table 2, which also includes data from related compounds from the literature. 

The structures confirm the piano stool type coordination geometry around the iron, in which one of the carbonyl ligands in the precursor complexes [FeX(Cp)(CO)_2_] is substituted for the phosphoramidite ligand **1b**. The bond angles around iron range from 90.76(8)° for the C(1)-Fe-P angle between the phosphoramidite and the carbonyl ligand and 95.58(8)° for the C(1)-Fe-I angle between the carbonyl and the iodo ligand. Thus, the coordination geometry of the complexes is best described as slightly distorted octahedral. For complex **4a**, an X-ray structure could also be determined. Unfortunately, despite multiple attempts, we were unable to obtain crystals of high quality and due to the poor crystal quality and weak diffraction, the resulting structure of **4a** was not of high quality. However, it establishes the connectivities for complex **4a**. Details of the structure determination and parameters as well as a graphical representation for **4a** are given in the Supplementary material (Table S1 and Figure S1). 

**Table 1:** Crystal data and structure refinement for **4b** and **5b**.

Empirical formula	C_40_H_31_BrFeNO_3_P (CH_2_Cl_2_)_2_	C_40_H_31_FeINO_3_P (C_6_H_14_)
Formula weight	910.24	873.55
Temperature, Wavelength	100(2) K, 0.71073 Å	100(2) K, 0.71073 Å
Crystal system, Space group	Orthorhombic, P2_1_2_1_2_1_	Orthorhombic, P2_1_2_1_2_1_
Unit cell dimensions	a = 10.3609(5) Å	a = 10.2311(8) Å
	b = 17.6708(8) Å	b = 14.9124(11) Å
	c = 21.2889(10) Å	c = 26.1055(19) Å
	α = β = γ = 90 °	α = β = γ = 90 °
Volume, Z	3897.7(3) Å^3^, 4	3982.9(5) Å^3^, 4
Density (calculated)	1.551 Mg/m^3^	1.457 Mg/m^3^
Absorption coefficient	1.769 mm^−1^	1.236 mm^−1^
Crystal size	0.57 x 0.13 x 0.08 mm^3^	0.31 x 0.07 x 0.06 mm^3^
Theta range for data collection	1.50 to 26.78°	2.07 to 24.99°
Reflections collected	40286	56634
Independent reflections	8268 [R(int) = 0.0520]	7016 [R(int) = 0.1303]
Absorption correction	Semi-empirical from equivalents	Semi-empirical from equivalents
Max. and min. transmission	0.8714 and 0.4316	0.9340 and 0.7044
Data / restraints / parameters	8268 / 0 / 478	7016 / 72 / 472
Goodness-of-fit on F2	1.02	1.06
Final R indices [I>2sigma(I)]	R1 = 0.0304, wR2 = 0.0555	R1 = 0.0566, wR2 = 0.1052
R indices (all data)	R1 = 0.0444, wR2 = 0.0593	R1 = 0.0922, wR2 = 0.1176
Absolute structure parameter	0.003(5)	0.04(3)
Largest diff. peak and hole	0.553 and -0.353 e.Å^−3^	0.993 and -0.932 e.Å^−3^

**Table 2:** Key bond lengths (Å) and angles (°).

	Complex 4b (X=Br, Y=1)	5b (X=I, Y=6)	6 (X=Br)	7 (X=Br)	8 (X=I)
X-Fe	2.4399(5)	2.5992(13)	2.437(0)	2.433	2.605(2)
Fe-C(Y)	1.778(3)	1.770(10)	1.744(3)	1.740	1.764(6)
C(Y)-O(1)	1.118(3)	1.149(10)	1.136(4)	1.195	1.077(7)
Fe-P	2.1501(7)	2.150(3)	2.163(1)	2.201	2.149(2)
P-N	1.642(2)	1.632(7)	-	-	
C(Y)-Fe-P	90.76(8)	91.1(3)	95.0(1)	95.20	92.9(2)
C(Y)-Fe-X	93.17(9)	89.5(3)	96.02	91.47	92.8(2)
P-Fe-X	92.03(2)	95.58(8)	91.73(3)	96.20	93.3(1)
O(1)-C(Y)-Fe	176.5(2)	177.8(7)	176.06	170.20	177.3(7)
N-P-Fe	121.54(8)	121.0(3)	-	-	-
O(3)-P-O(2)	100.36(9)	100.0(3)	104.32	-	-

The molecular structures of **4a**, **4b** and **5b** do not show significant differences. However, the C(1)-Fe-Br angle for **4b** of 93.17(9)° is larger than that one of 89.5(3)° for the C(6)-Fe-I angle in **5b**. In turn, the P-Fe-Br angle for **4b** of 92.03(2)° is smaller than that one of 95.58(8)° for the P-Fe-I angle in **5b**. It appears that the larger iodo ligand in **5b** causes a larger angle between the iodo and the phosphoramidite ligand, which, in turn, renders the iodo and the carbonyl ligand closer together, resulting in a smaller angle. 

**Figure 4:**
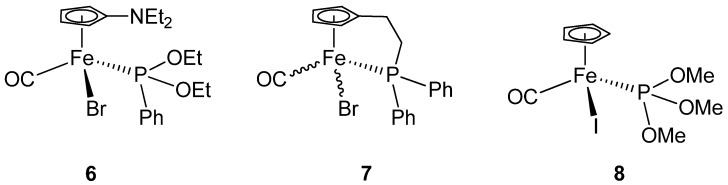
Structurally related pianostool type iron complexes.

In order to analyze the influence of the phosphoramidite ligand on the structure of the complex, we compared key structural data with other complexes of the general formula [FeX(Cp)(CO)L], where L is a phosphorus donating ligand and X either Br or I. Structurally characterized complexes of this formula are rare, and three examples **6** [44], **7** [50] and **8** [51] from the literature are displayed in Figure 4. The available structural parameters are listed in Table 2. 

It can be seen that the five complexes do not significantly differ structurally and bond lengths and angles are comparable. Only complex **7** differs slightly from complexes **4b** and **5b** with respect to the Fe-P bond length. The Fe-P bond length for **4b** is 2.1501(7) Å and for **5b** it is 2.150(3) Å. These bond lengths are, thus, slightly shorter than in complex **7** (2.201 Å). This trend can be explained if it is assumed that the phosphoramidite ligand in **4b** and **5b** allows for more back bonding than the PPh_2_R ligand in **7**. Stronger backbonding from the metal to the ligand strengthens and consequently shortens the Fe-P bond in **4b**.

### 2.2. Application of the New Iron Phosphoramidite Complex 4a in Catalysis

We were next interested to determine if the new iron phosphoramidite complexes **4a **and **4b** are useful as catalysts in organic oxidation reactions. The original driving force to apply chiral iron complexes was to investigate potential alkane oxidations to obtain chiral alcohols. Screening experiments are compiled in Table 3. Activated methylene groups in such substrates as cinnamyl alcohol, tetrahydronaphthalene, fluorene, diphenylmethane and dihydroanthracene could be oxidized to the corresponding ketones catalyzed by iron complex **4a** using *t*-BuOOH as the oxidant. Less activated methylene or methyl groups in such substrates as toluene, adamantane or cyclooctene were not oxidized under the reaction conditions in Table 3. While *t*-BuOOH worked well as oxidant, H_2_O_2_, ethaneperoxoic acid and 3-chlorobenzoperoxoic acid (mCPBA) typically gave no oxidations, with the exception of the oxidation of cinnamyl alcohol. Somewhat unexpectedly, in this case an oxidative cleavage of the double bond to give benzaldehyde was also observed. Pyridine was determined to be the solvent of choice, and in most cases the reactions were performed at room temperature. Ketones were typically the only reaction products; alcohols were not observed by GC/MS. *Significantly, the precursor complexes* [FeX(Cp)(CO)_2_] (**2**, **3**) *showed much slower rates in the oxidation reactions* (entries 17 to 20 in Table 3). No reactions were observed in the absence of the catalyst, except for the oxidation of cinnamyl alcohol (slow and incomplete oxidation) and tetrahydronaphthalene-1-ol (entries 2 and 6 in Table 3). The catalytic performance of complexes **4b** and **5b** were comparable, but complex **4a** was used for optimizations and further studies, as its ligand **1a** is synthetically more easily accessed than ligand **1b**.

**Table 3:** Screening of catalyst activity. 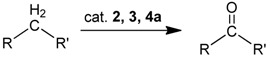

Entry	Substrate	Oxidant ^a^	Time / Temperature	Catalyst Loading	Solvent	Product	Yield (%) ^b^
1	toluene	*t*-BuOOH	48 h / 90 °C	2 mol% **4a**	pyridine	NR ^e^	
2	cinnamyl alcohol	*t*-BuOOH	24 h / 80 °C	–	acetonitrile	cinnamaldehyde	30
3	cinnamyl alcohol	H_2_O_2_	24 h / rt	10 mol% **4a**	CH_2_Cl_2_	benzaldehyde	100
4	cinnamyl alcohol	*t*-BuOOH	24 h / rt	10 mol% **4a**	pyridine	cinnamaldehyde benzaldehyde	80 ~20
5	tetrahydro-naphthalene	*t*-BuOOH	18 h / 90 °C	2 mol% **4a**	pyridine	tetrahydronaph-thalene-1-one	100
6	tetrahydronaph-thalene-1-ol	*t*-BuOOH	16 h / rt	–	pyridine	tetrahydronaph-thalene-1-one	100
7	diphenyl-methane	*t*-BuOOH	36 h / 82 °C	2 mol% **4a**	pyridine	benzophenone	100
8	fluorene	*t*-BuOOH	36 h / rt	2 mol% **4a**	pyridine	fluorenone	100
9	fluorene	mCPBA ^c^	36 h / rt	2 mol% **4a**	pyridine	fluorenone	traces
10	fluorene	CH_3_COOOH ^d^	36 h / rt	2 mol% **4a**	pyridine	fluorenone	traces
11	fluorene	*t*-BuOOH	36 h / rt	2 mol% **4a**	pyridine	fluorenone	100
12	dihydro-anthracene	*t*-BuOOH	36 h / rt	2 mol% **4a**	pyridine	anthraquinone	100
13	adamantane	*t*-BuOOH	36 h / rt	2 mol% **4a**	pyridine	NR ^e^	
14	adamantane	*t*-BuOOH	36 h / 90 °C	2 mol% **4a**	pyridine	NR ^e^	
15	diphenyl-methane	H_2_O_2_	36 h / rt	10 mol% **4a**	CH_2_Cl_2_	NR ^e^	
16	cyclooctene	*t*-BuOOH	42 h / rt	2 mol% **4a**	pyridine	NR ^e^	
17	fluorene	*t*-BuOOH	36 h / rt	2 mol% **2**	pyridine	fluorenone	30
18	dihydro-anthracene	*t*-BuOOH	36 h / rt	2 mol% **2**	pyridine	anthraquinone	9 ^f^
19	diphenyl-methane	*t*-BuOOH	36 h / rt	2 mol% **2**	pyridine	benzophenone	27
20	fluorene	*t*-BuOOH	36 h / rt	2 mol% **3**	pyridine	fluorenone	21

^a^ Oxidants were applied in 3.0 fold excess.^b ^Determined by GC/MS. ^c^ 3-chlorobenzoperoxoic acid.^d^ ethaneperoxoic acid.^e^ No reaction. Only starting material was detected, and at most trace quantities of oxidation products. ^f^ 59% anthracene were detected by GC/MS.

Under optimized reaction conditions, complex **4a** was then utilized in the oxidation of a variety of substrates, as compiled in Table 4. Diphenylmethane, fluorene, dihydroanthracene, cinnamyl alcohol and phenylmethanol were oxidized to the corresponding aldehydes or ketones in 80 to 31% yields. About three equivalents of *t*-BuOOH with a catalyst loading of 2 mol% in pyridine as solvent were employed. The turnover frequencies ranged from 0.37 to 1.39 h^–1^.

**Table 4:** Iron catalyzed oxidation reactions. 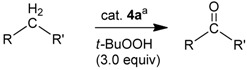

Entry	Starting Material	Product	Yield ^b^	TOF / h^−1 c^
1	diphenylmethane	benzo-phenone	56%	0.78
2	fluorene	fluorenone	80%	1.11
3	dihydroanthracene	anthra-quinone	54% ^d^	1.39
4	cinnamylalcohol	cinnamyl aldehyde	*31*% ^e^	*0.37* ^e^
5	phenylmethanol	benzaldehyde	*47*% ^e^	*0.56* ^e^

^a^ Conditions: substrate (0.602 mmol), *t*-BuOOH in decane (1.8 mmol), catalyst **4a** (2 mol%), 36 h in pyridine (1 mL) at rt. ^b^ Isolated yields after column chromatography. ^c^ Turnover frequency determined from isolated yield: number of moles (product) over number of moles (catalyst) times reaction time. ^d^ The product contained ca. 10% anthracene (as assessed by ^1^H-NMR). ^e^ NMR yields from reactions run in pyridine-*d*_5_; it was not possible to separate the products from the decane (which is the solvent for the *t*-BuOOH employed in the reaction). The TOF was calculated from the NMR data.

### 2.3. Further Experiment to Better Understand the Oxidation Reactions

**Figure 5:**
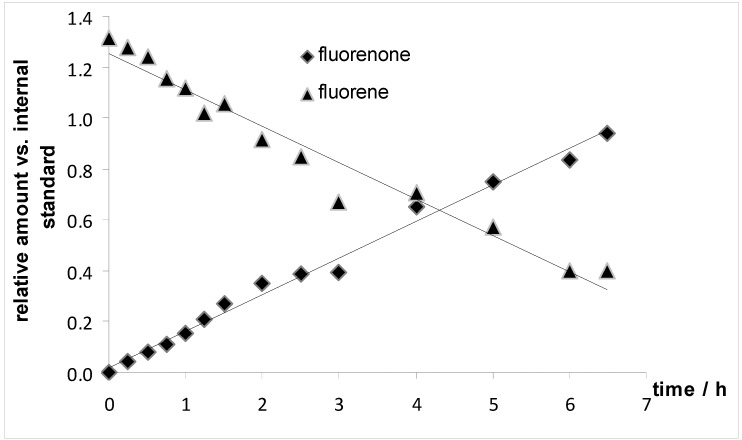
Monitoring of substrate decay and product formation over time.

To obtain further details of the oxidation reactions, additional experiments were performed. The oxidation of fluorene catalyzed by **4a** was monitored by GC for the first seven hours (entry 2 in Table 4). Figure 5 shows a plot of the formation of fluorenone and the consumption of fluorene *versus* time. The reaction appears to be pseudo zero order with respect to the substrate fluorene and fluorenone formation. However, first order reactions might very well appear zero order in their initial stage. The observed rate constant derived from the slope of the linear trend from the fluorene consumption and the fluorenone formation in Figure 5 was calculated to be 0.14 h^−1^. No induction period was observed for the reaction. 

As the complex **4a** is coordinatively saturated, an open coordination site from ligand loss is required for catalytic activity. Proton and ^31^P-NMR spectra of the metal complex in pyridine-*d*_5_ showed no decomposition after 24 h, suggesting that the pyridine solvent applied in the oxidation reactions in Table 4 does not displace ligands. To obtain insight into the catalytically active species generated in the reaction mixture, the catalyst **4a** and the oxidant *t*-BuOOH were combined in the absence of substrate in pyridine-*d*_5_. After 24 h, NMR (^1^H, ^31^P), IR and MS spectra were recorded for the residue after solvent removal. In the ^31^P-NMR, a peak around 14 ppm was observed, which we tentatively assigned to the oxidized phosphoramidite ligand **1a**. In the ^1^H spectrum, two singlets around 2.7 ppm for the NC*H*_3_ groups were observed, which are significantly shifted compared to their resonances in the metal complex **4a** (2.85 ppm). The MS spectrum of the residue showed a peak for ligand **1a** plus oxygen, suggesting that the loss of the phosphoramidite ligand by oxidation might be one step towards the formation of the catalytically active species in the reaction mixture. This also could explain, why [FeBr(Cp)(CO)_2_] (**2**) and [Fe(Cp)I(CO)_2_] (**3**) are much less active relative to **4a** (Table 3, entries 17 to 20), because the complexes do not contain ligands that can be oxidatively removed. Furthermore, after oxidation the absorption for the terminal CO ligand in **4a** in the IR spectrum disappeared, and instead, a new band at 1713 cm^−1^ was observed, which could be evidence for a carbonyl bridged dimeric iron species.

**Scheme 2:**
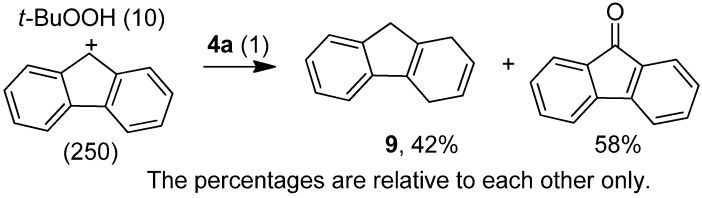
Experiment employing an excess of substrate over the oxidant.

As mentioned above, no alcohols were observed as the reaction products (Table 3 and Table 4). To avoid overoxidation, some authors employ a large excess of the substrate over the oxidant in test reactions to elucidate a mechanism [52]. Accordingly, we performed a reaction with a 25-fold excess of the substrate fluorene relative to the oxidant *t*-BuOOH in the presence of the iron catalyst **4a** (250:10:1 substrate:*t*-BuOOH:**4a**, Scheme 2). Under these reaction conditions, the alcohol product was not observed by GC/MS. Instead, the hydrogenated species **9** and the corresponding ketone product were detected in a 42:58 ratio. 

**Scheme 3:**
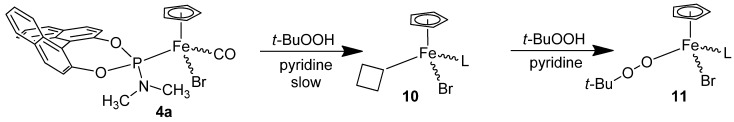
Potential formation of the catalytically active species in the oxidation reactions. The square denotes an open coordination site.

The mechanism of iron-catalyzed oxidation reactions utilizing H_2_O_2_ strongly depends on the reaction conditions, the solvent, the oxidant and the relative ratio of the reactants [53]. Sawyer proposed a mechanism which he termed “Oxygenated Fenton Chemistry” [40]. Barton published a series of iron catalyst systems (Gif chemistry) for alkane oxidation with peroxides [41,54] and suggested a non-radical mechanism. However, some authors suggested that free radicals were involved [55,56] while others corroborate Barton’s non-radical mechanism [57]. Iron catalyzed reactions utilizing *t*-BuOOH appear to follow a radical mechanism [53]. The formation of a peroxo species [Fe-OO*t*-Bu] [58,59] is often considered to be the first step in the oxidation reactions with *t*-BuOOH which gives an LMCT absorption between 500 and 600 nm in UV-vis spectra [60,61]. The peroxo species [Fe-OO*t*-Bu] can undergo homolytic bond cleavage to form either *t*-BuO℘ or *t*-BuOO℘radicals and oxoiron species Fe=O, which are the actual oxidants [60]. The peroxo species [Fe-OO*t*-Bu] could not be observed by UV when complex **4a** was combined with *t*-BuOOH in the absence of substrates. Unfortunately, due to the formation of unidentified species with strong absorptions around 300 nm, the reaction of the iron complex with *t*-BuOOH could not be monitored over extended periods of time by UV-vis, as performed by other authors [60]. We thus do not have spectroscopic evidence that the intermediate [Fe-OO*t*-Bu] forms during the reaction but its formation might be slow in our case (as are the oxidation reactions in Table 4). Based on our data we cannot definitely establish a mechanism for our oxidation reactions. It appears reasonable that slow oxidative removal of the phosphoramidite ligand creates species **10** with an open coordination side (Scheme 3), which then further reacts to afford the peroxo complex [Fe-OO*t*-Bu] (**11**). Based on our spectroscopic data, the exact nature of complexes **10** and **11** cannot be established, either these are pyridyl coordinated species or carbonyl bridged dimers. Cleavage of the peroxo bridge in **11** might provide the oxidants, and its slow formation would explain the long reaction times and the kinetics in substrate and product for the oxidation of fluorene (Figure 5). 

Thus, placing a phosphoramidite ligand in the coordination sphere of a complex of the general formula [FeBr(Cp)(CO)L] is a strategy to keep a steady supply of the catalytically active species **11** in the reaction mixture, slowing catalyst and peroxide decomposition pathways. Thus, slow addition of the peroxide oxidant to the reaction mixture is not necessary, as performed by other authors [62].

## 3. Experimental

### 3.1. General

Chemicals were treated as follows: acetonitrile and pentane were distilled from CaH_2_. Toluene was distilled from Na/benzophenone. Other solvents: CHCl_3_, pyridine, CH_2_Cl_2_, hexanes, the substrates for the catalytic experiments (Aldrich), and *t*-BuOOH (5.5 M in decane, Fluka) were used as received. “(*R*)-BINOL-*N,N*-dimethylphosphoramidite” **1a** [63] “(*R*)-BINOL-*N,N*-dibenzylphosphoramidite” **1b**, [64] “(*R*)-BINOL-*N,N*-diisopropylphosphoramidite” **1c** [64], [FeBr(Cp)(CO)_2_] (**2**) [42] and [Fe(Cp)I(CO)_2_] (**3**) [47] were synthesized according to the literature. Metal complex syntheses were carried out under argon employing standard Schlenk techniques. Workup as well as the catalytic experiments were performed under aerobic conditions. 

The NMR spectra were obtained at room temperature on either a Bruker Avance 300 MHz or a Varian Unity Plus 300 MHz instrument and were referenced to a residual solvent signal. All assignments are tentative. GC/MS spectra were recorded on a Hewlett Packard GC/MS system model 5988A. Exact masses were acquired on a JEOL MStation [JMS-700] mass spectrometer. IR spectra were recorded on a Thermo Nicolet 360 FT-IR spectrometer. Elemental analyses were performed by Atlantic Microlab Inc., Norcross, GA, USA.

CCDC 727527 (**4b**), CCDC 727535 (**4a)** and CCDC 765699 (**5b**) contain the supplementary crystallographic data for this paper. These data can be obtained free of charge via www.ccdc.cam.ac.uk/conts/retrieving.html (or from the CCDC, 12 Union Road, Cambridge CB2 1EZ, UK; Fax: +44 1223 336033; E-Mail: deposit@ccdc.cam.ac.uk). 

### 3.2. Synthesis of “[FeBrCp(CO)(1a)]”, **4a**

A Schlenk flask was charged with phosphoramidite **1a** (0.078 g, 0.304 mmol) and [FeBr(Cp)(CO)_2_] (**2**, 0.120 g, 0.334 mmol), and dry toluene (10 mL) was added. The solids dissolved and the light red colored solution was then heated at 90 °C under an atmosphere of argon for 3 h. The color of the solution darkens. The solvent was removed and the greenish solid was washed with dry pentane (2 × 4 mL). The solid was dissolved in chloroform and re-precipitated by adding hexanes. The solvents were decanted and the residue was dried for two days under high vacuum to give the complex **4a** (0.061 g, 0.104 mmol, 34%) as a greenish solid, which was isolated as a mixture of diastereomers (77:23, as assessed by ^1^H and ^31^P). Calcd for C_28_H_23_BrPFeNO_3_: C, 57.17; H, 3.94. Found: C, 52.38; H, 3.85 [65].

HRMS calcd for C_28_H_23_^79^BrNO_3_P^54^FeNa, 609.98456; found, 609.9854 [66]. MS (FAB, 4-NBA + NaI) [67] *m*/*z* 610 ([**4a**+Na]^+^, 10%) [66], 559 ([**4a**–CO]^+^, 15%), 480 ([**4a**–Br–CO]^+^, 30%). IR (cm^–1^, neat solid) ν_C__≡__O_ 1971 (s, sh). 

To separate the diastereomers, the crude reaction mixture was allowed to cool to rt, and a solid precipitated. The solution was decanted from the solid. The solid was washed with dry diethyl ether (about 2 × 2 mL). The solid was dried under vacuum for 2 days at 50 °C, yielding **4a** (0.052 g, 0.09 mmol, 26%) as 50:50 mixture of diastereomers (^1^H, ^13^C, ^31^P NMR).

From the decanted solution, the solvent was removed by vacuum. The residual solid was dissolved in CH_2_Cl_2 _(4 mL) and the solvent was allowed to slowly evaporate, causing precipitation of a solid. The remaining solvent was decanted, and the solid was washed with hexanes (4 mL). The solid was dried under high vacuum at 50 °C for 2 days to yield a greenish colored solid of **4a** (0.050 g, 0.09 mmol, 26%) as a mixture 85:15 mixture of diastereomers (^1^H-, ^31^P-NMR). 

Based on the intensities of the signals in the NMR spectra, the data for the two isomers are given below separately. 

NMR (δ, CD_2_Cl_2_, major diastereomer): ^1^H: 8.05−7.95 (m, 5H, binapthyl), 7.38−7.30 (m, 9H, binapthyl), 7.18−7.16 (d, 1H, 4.69, *J*_HH_ = 6.0 Hz), 4.77 (d, 5H, *J*_HH_ = 1Hz, Cp), 2.91 (s, 3H, NC*H*_3_), 2.87 (s, 3H, NC*H*_3_’); ^13^C{^1^H}: 217.4 (d, *J*_CP_ = 44.7 Hz, *C*O), 150.3, 150.2, 148.2 (d, *J*_CP_ = 5.4 Hz), 133.2 (d, *J*_CP_ = 1.6 Hz), 132.8 (d, *J*_CP_ = 1.6 Hz), 131.7, 131.6 (d, *J*_CP_ = 1.2 Hz), 130.9 (d, *J*_CP_ = 1.5 Hz), 130.5, 128.7, 128.5, 127.2, 126.8, 126.6 (d, *J*_CP_ = 4.2 Hz), 125.7, 125.4, 123.6 (d, *J*_CP_ = 2.6 Hz), 123.0 (d, *J*_CH_ = 2.6 Hz), 121.6 (d, *J*_CP_ = 2.6 Hz), 121.3 (d, *J*_CP_ = 1.9 Hz, aromatic), 82.9 (d, *J*_CP_ = 1.9 Hz, Cp), 38.8 (s, N*C*H_3_), 38.7 (s, N*C*H_3_’); ^31^P {^1^H}: 198.5 (s). 

NMR (δ, CD_2_Cl_2_, minor diastereomer, partial) [68]: ^1^H 4.73 (d, 5H, *J*_HP_ = 1.0 Hz, Cp), 2.69 (s, 3H, NC*H*_3_), 2.65 (s, 3H, NC*H*_3_’);^ 13^C{^1^H}: 131.5, 130.3, 128.6, 127.5, 127.1, 126.4, 124.3, 124.1, 123.3 (aromatic), 83.1 (d, *J*_CP_ = 1.5 Hz, Cp), 38.62 (s, N*C*H_3_), 38.58 (s, N*C*H_3_’); ^31^P {^1^H}: 200.4 (s).

### 3.3. Synthesis of “[FeBrCp(CO)(1b)]”, **4b**

A Schlenk flask was charged with phosphoramidite **1b** (0.132 g, 0.258 mmol) and [FeBr(Cp)(CO)_2_] (**2**, 0.060 g, 0.234 mmol), and dry toluene (10 mL) was added. The solids dissolved and the light red colored solution was then heated at 90 °C under an atmosphere of argon for 3 h. The color of the solution darkened. Upon cooling, the solvent was removed under high vacuum. The brownish solid was washed with dry pentane (about 2 × 4 mL) and then dissolved in 4 mL of CH_2_Cl_2_ and layered with hexanes and stored at −18 °C. A precipitate formed, and the mother liquor was removed and the residual solid was washed with hexanes. The crystallite solid was dried under vacuum (oil pump) for two days at 40 °C to give the complex **4b** as tan solid (0.125 g, 0.17 mmol, 65%) as a mixture of diastereomers (90:10, as assessed by ^1^H). Calcd for C_40_H_31_FeBrPO_3_N: C, 64.18; H, 4.22. Found: C, 66.28; H, 4.62 [65]. 

HRMS calcd for C_40_H_31_^79^BrNO_3_P^56^FeNa, 762.04706; found, 762.0475 (FAB+). MS (FAB, 4-NBA + NaI) [67] *m*/*z* 764 ([4b+Na]^+^, 10%) [66], 713 ([**4b**–CO]^+^, 25%), 632 ([**4b**–CO–Br]^+^, 35%), 534 ([**1b**+Na]^+^, 95%); IR (cm^–1^, neat solid) ν_C≡O_ 1978 (s).

NMR (δ, CD_2_Cl_2_) [68] ^1^H: 8.13−8.00 (m, 2H, aromatic), 7.88-7.74 (m, 2H, aromatic), 7.53−7.16 (m, 21H, aromatic), 5.13−5.03 (m, 2H, NC*H*_2_), 4.87 (s, 5H, Cp), 4.75 (s, 0.5H, Cp’, minor diastereomer), 4.65−4.50 (m, 0.2H, NC*H*_2_’, minor diastereomer), 3.80−3.65 (m, 0.2H, NC*H*_2_’, minor diastereomer), 3.54−3.45 (m, 2H, NC*H*_2_); ^13^C{^1^H}: [68] 216.9 (d, *J*_CP_ = 45.3 Hz, *C*O), 150.2, 150.0, 148.1 (d, *J*_CP_ = 4.9 Hz), 137.4 (d, *J*_CP_ = 2.7 Hz), 133.3, 132.6, 131.8, 131.4, 130.8, 130.5, 129.6, 128.8, 128.6, 128.6, 126.5, 127.9, 127.7, 127.3, 126.8 (d, *J*_CP_ = 3.5 Hz), 126.5, 125.8, 125.4, 123.9, 122.7, 121.5, 121.2 (aromatic), 83.3 (d, *J*_CP_ = 1.5 Hz, Cp), 83.0 (d, *J*_CP_ = 1.6 Hz, Cp’, minor diastereomer), 49.9 (d, *J*_CP_ = 6.5 Hz, *C*H_2_); ^31^P{^1^H}: 196.3 (s, major diastereomer). 

### 3.4. Attempted synthesis of “[FeBrCp(CO)(1c)]”, **4c**

To a Schlenk flask containing phosphoramidite **1c** (0.100 g, 0.251 mmol) and [FeBr(Cp)(CO)_2_] (**2**, 0.056 g, 0.219 mmol), dry toluene (10 mL) was added. The solids dissolved and the light red colored solution was then heated at 90 °C under an atmosphere of argon for 5 h. The color of the solution darkens. Upon cooling, the solvent was removed under high vacuum. The light green colored solid was washed with dry pentane (about 2 × 4 mL). The solid was then dried under vacuum (oil pump) for two days to give a greenish solid as a mixture of phosphoramidite **1c** and the complex **4c** as single diastereomer (0.079 g recovered mass, ca. 60% spectroscopic purity for **4c**, as assessed by ^1^H and ^31^P). Only the analytical data for **4c** is given below. 

HRMS calcd for C_32_H_31_^79^BrNO_3_P^56^FeNa, 666.04712; found 666.0443 (FAB+). MS (FAB, 4-NBA) [67] *m*/*z* 644 ([**4c**]^+^, 5%), 615 ([**4c**–CO]^+^, 100%), 536 ([**4c**–CO–Br]^+^, 75%), 416 ([**1c**+H]^+^, 45%); IR (cm^–1^, oil film) ν_C≡O_ 1976 (s).

NMR (δ, CD_2_Cl_2_) [68] ^1^H: 8.33−6.87 (m, 18H, aromatic), 4.97 (s, 5H, Cp), 4.66 (m, 2H, C*H*), 1.68 (d, 6H, *J*_HH_ = 6.6 Hz, C*H*_3_), 1.21 (d, 6H, *J*_HH_ = 6.2 Hz, C*H*_3_’); ^13^C{^1^H}: 151.5, 151.3, 148.9, 134.6, 134.1, 132.9, 132.4, 131.5, 131.3, 129.7, 129.6, 128.4, 127.9, 127.7, 127.5, 126.8, 126.3, 125.0, 123.9, 123.1 (aromatic), 83.9 (Cp), 49.7 (d, *J*_CP_ = 7.2 Hz, *C*HCH_3_), 24.6 (*C*H_3_); ^31^P{^1^H}: 199.9 (s).

### 3.5. Synthesis of “[Fe(Cp)I(CO)(1b)]”, **5b**

A Schlenk flask was charged with phosphoramidite **1b** (0.185 g, 0.362 mmol) and [Fe(Cp)I(CO)_2_] (**3**, 0.100 g, 0.329 mmol), and hexane (10 mL) was added. The flask was fitted with reflux condenser. The suspension was heated to reflux and [CpFe(CO)_2_]_2_ (0.004 g, 0.011 mmol) was added to the reaction mixture. The color of the solution darkened almost instantaneously. The solution was refluxed for 30 minutes. Upon cooling, the reaction mixture was filtered through a short pad of cellulose, the pad was washed with hexanes (2 × 5 mL) and with CH_2_Cl_2_ (2 × 5 mL). The solvent was removed from the combined filtrates and the crystalline solid was dried under vacuum (oil pump) to give the complex **5b** as green solid (0.209 g, 0.265 mmol, 81%) as diastereomeric mixture (73:27 as assessed by ^1^H NMR). 

HRMS calcd for C_39_H_31_NO_2_P^56^FeI, 759.0487; found, 759.0486 (FAB+). MS (FAB, 4-NBA) *m/z* 788 ([**5b**+H]^+^, 10%), 759 ([**5b**–CO+H]^+^, 100%), 694 ([**5b**–CO–Cp+H]^+^, 25%), 633 ([**5b**-CO-I]^+^, 60%); IR (cm^−1^, neat solid) ν_C≡O_ 1971 (s).

NMR (δ, CD_2_Cl_2_, major isomer) ^1^H: 8.16 (d, 1H, aromatic, J_HH_ = 8.7 Hz), 8.05 (d, 1H, aromatic, *J*_HH_ = 8.3 Hz), 7.88 (d, 2H, aromatic, *J*_HH_ = 8.3 Hz), 7.83 (d, 2H, aromatic, *J*_HH_ = 9.2 Hz), 7.55−7.19 (m, 24H, aromatic), 5.11−5.04 (m, 2H, C*H*_2_), 4.90 (s, 5H, Cp), 3.55−3.46 (m, 2H, C*H*_2_); ^13^C{^1^H}: 217.7 (d, *J*_CP_ = 21.8 Hz, *C*O), 150.7, 150.5, 148.6 (d, *J*_CP_ = 5.3 Hz), 137.7 (d, *J*_CP_ = 2.8 Hz), 133.6 (d, *J*_CP_ = 1.5 Hz), 132.9 (d, *J*_CP_ = 1.5 Hz), 132.1, 131.7, 131.1, 130.8, 129.8, 129.2, 129.1, 128.9, 128.8, 128.2, 127.6, 127.2, 127.1, 126.9, 126.1, 125.8, 121.9, 121.6 (aromatic), 83.3 (Cp), 50.7 (d, *J*_CP_ = 6.8 Hz, *C*H_2_); ^31^P{^1^H}: 201.8 (s). 

NMR (δ, CD_2_Cl_2_, minor isomer) ^1^H: 4.79 (s, 0.8H, Cp), 4.74−4.66 (m, 0.4H, C*H*_2_), 3.76−3.61 (m, 0.6H, C*H*_2_); ^13^C{^1^H}: 82.9 (Cp), 51.2 (d, *J*_CP_ = 6.8 Hz, *C*H_2_); ^31^P{^1^H}: 203.2 (s).

### 3.6. Typical Procedure for the Catalytic Experiments

The substrate fluorene (0.100 g, 0.602 mmol) and the catalyst **4a** (0.007 g, 0.012 mmol) were dissolved in pyridine (1.0 mL). The oxidant *t*-BuOOH (0.33 mL of a 5.5 M solution in decane, 1.8 mmol) was added and the brownish solution was shaken for 36 h at room temperature. The pyridine was removed under vacuum. The product 9-fluorenone was isolated by column chromatography (silica gel; CH_2_Cl_2_) as yellow-crystalline solid (0.0872 g, 0.479 mmol, 80%). Analytical data and NMR spectra (^1^H, ^13^C) are given in the Supporting information. 

### 3.7. Monitoring of the Oxidation of Fluorene to Fluorenone over Time (Figure 5)

A screw capped vial was charged with fluorene (0.100 g, 0.602 mmol) and the catalyst **4a** (0.007 g, 0.012 mmol). Pyridine (1 mL) was added and the solids dissolved to give a clear yellow solution. *t*-BuOOH (0.33 mL of a 5.5 M solution in decane, 1.8 mmol) was then added in one portion and the reaction mixture was shaken at rt. For analysis, aliquots were taken from the reaction mixture, filtered through a short pad of alumina (which was washed with 2 mL CH_2_Cl_2_), and injected into the GC/MS instrument. The substrate decay and product formation over time was determined by the ratio of its signal intensity to the signal intensity of decane (which is the solvent for *t*-BuOOH and served as an internal standard). 

### 3.8. Reaction of the Catalyst 4a with the Oxidant t-BuOOH without Substrate.

A vial was charged with catalyst **4a** (0.062 g, 0.011 mmol), and pyridine (0.5 mL) was added. To the solution, *t*-BuOOH (0.150 mL of a 5.5 M solution in decane, 0.831 mmol) was added in one portion and the solution was stirred for 24 to 36 h. The pyridine was removed under vacuum, and the solid residue was analyzed by MS and NMR (^1^H, ^31^P).

MS (FAB, 4-NBA) *m/z* 376 ([**1a**+O]^+^, 40%); MS (FAB, 4-NBA + NaI) *m/z* 398 ([**1a**+O+Na]^+^, 55%); IR (cm^–1^, neat solid) ν_C≡O_ 1713 (m). NMR (δ, CDCl_3_, partial) ^1^H: 8.01−7.94 (m, 4H, binapthyl), 7.60−7.30 (m, 8H, binapthyl), 2.70 (d, 6H, *J*_HH_ = 9.61 Hz); ^31^P{^1^H}: 14.9 (s).

### 3.9. X-ray Structure Determination for 4b and 5b

X-ray quality crystals of **4b** and **5b** were obtained by layering a CH_2_Cl_2_ solution with hexanes, which was stored at –18 °C for one to four weeks. Crystals were mounted from Paratone oil to a Bruker Kappa Apex II single crystal X-Ray diffractometer equipped with an Oxford Cryostream LT device. Intensity data were collected by a combinations of ϖ and φ scans. Apex II, SAINT and SADABS software packages (Bruker Analytical X-Ray, Madison, WI, 2008) were used for data collection, integration and correction of systematic errors, respectively. 

Crystal data and intensity data collection parameters are listed in Table 1. Structure solution and refinement were carried out using the SHELXTL- PLUS software package [69]. The structures were solved by direct methods and refined successfully in the space group P2_1_2_1_2_1_. The non-hydrogen atoms were refined anisotropically to convergence. All hydrogen atoms were treated using appropriate riding model (AFIX m3). Complete listings of positional and isotropic displacement coefficients for hydrogen atoms, anisotropic displacement coefficients for the non-hydrogen atoms and tables of calculated and observed structure factors are available in electronic format.

## 4. Conclusions

In summary, we have synthesized the first iron complexes of the general formula [FeX(Cp)(CO)(**1**)] (X=Br, I) containing common phosphoramidite ligands **1**. The new complexes are chiral at the metal, and were obtained with diastereomeric excesses between 73:27 and 90:10. The new iron complexes are catalyst precursors in the oxidation of activated, benzylic methylene groups with *t*-BuOOH to give exclusively the corresponding ketones in 31−80% isolated yields. This is the first application of iron phosphoramidite complexes as catalyst precursors, and the work described herein sets the stage for further catalytic applications of this class of complexes. 

## Supplementary Materials

Supplementary data (experimental details and NMR data and NMR spectra for the catalysis products in Table 3, NMR spectra of the complexes **4a** and **4b** and **5b**, X-ray structure determination of **4b**) associated with this article can be found at http://www.mdpi.com/1420-3049/15/4/2631/S1.
