# Supplementary material for: New Chiral Phosphoramidite Complexes of Iron as Catalytic Precursors in the Oxidation of Activated Methylene Groups
Source: Molecules. 2010 Apr 12;15(4):2631–50. doi: 10.3390/molecules15042631 (PMC6257337; doi:10.3390/molecules15042631)
Supplement: Supplementary file 1 [file molecules-15-02631-s001.pdf]

Molecules **2010**, *15*, 1-x manuscripts; doi:10.3390/molecules150x000x

OPEN ACCESS

molecules

ISSN 1420-3049

www.mdpi.com/journal/molecules

Supplementary file

Novel Chiral Phosphoramidite Complexes of Iron and their Catalytic Activity in the Oxidation of Activated Methylene Groups

Pushkar Shejwalkar [1], Nigam P. Rath [2] and Eike B. Bauer [3,*]

[1] University of Missouri - St. Louis, Department of Chemistry and Biochemistry, One University Boulevard, St. Louis, MO 63121, USA; E-Mail: pssgk8@umsl.edu

[2] Department of Chemistry and Biochemistry and Center for Nanoscience, University of Missouri - St. Louis, One University Boulevard, St. Louis, MO 63121, USA; E-Mail: rathn@umsl.edu

[3] University of Missouri - St. Louis, Department of Chemistry and Biochemistry, One University Boulevard, St. Louis, MO 63121, USA; E-Mail: bauere@umsl.edu

* Author to whom correspondence should be addressed; E-Mail: bauere@umsl.edu; Tel.: +1-001-314-516- 5311; Fax: +1-001-314-516-5342.

Catalysis experiments in Table 4 1
X-Ray determination for Complex **4a** 2
^{1}H and ^{13}C NMR spectra of complexes **4a**, **4b** and **5b** 4
^{1}H and ^{13}C NMR spectra of the catalysis experiments in Table 4 7
References 11

Catalysis experiments in Table 4

The substrate fluorene (0.100 g, 0.602 mmol) and the catalyst **4a** (0.007 g, 0.012 mmol) were dissolved in pyridine (1.0 mL). The oxidant *t*-BuOOH (0.33 mL of a 5.5 M solution in decane, 1.8 mmol) was added and the brownish solution was shaken for 36 h at rt. Pyridine was removed under vacuum. The product 9-fluorenone was isolated by column chromatography, as yellow-crystalline solid (0.087 g, 0.479 mmol, 80%) using CH_2Cl_2 as eluent.

NMR (δ, $CDCl_3$) 7.57 (d, 2H, aromatic), 7.44-7.37 (m, 4H, aromatic), 7.25-7.18 (m, 2H, aromatic); $^{13}C\{^1H\}$ 194.1 (s, *C*O), 144.7 (s, aromatic), 134.9 (s, aromatic), 134.4 (s, aromatic), 129.3 (s, aromatic), 124.5 (s, aromatic), 120.5 (s, aromatic); IR (cm^{-1}, neat liquid) $\nu_{C\equiv O}$ 1713 (s), 1611 (s), 1599 (s).

The substrate diphenylmethane (0.100 g, 0.595 mmol) and the catalyst **4a** (0.007 g, 0.012 mmol) were dissolved in pyridine (1.0 mL). The oxidant *t*-BuOOH (0.65 mL of a 5.5 M solution in decane, 3.6 mmol) was added and the brownish solution was shaken for 36 h at rt. Pyridine was removed under vacuum. The product benzophenone was isolated by column chromatography, as colorless liquid (0.061 g, 0.334 mmol, 56%) using CH_2Cl_2 as eluent.

NMR (δ, acetone-d_6) 7.82-7.76 (m, 2H, aromatic), 7.68-7.63 (m, 4H, aromatic), 7.58-7.52 (m, 2H, aromatic); $^{13}C\{^1H\}$ 197.1 (s, *C*O), 139.2 (s, aromatic), 133.8 (s, aromatic), 131.1 (s, aromatic), 129.8 (s, aromatic); IR (cm^{-1}, neat liquid) $\nu_{C\equiv O}$ 1656 (s), 1598 (m), 1577 (w), 1274 (s).

The substrate 9,10-dihydroanthracene (0.100 g, 0.555 mmol) and the catalyst **2a** (0.006 g, 0.011 mmol) were dissolved in pyridine (1.0 mL). The oxidant *t*-BuOOH (0.3 mL of 5.5M solution in decane, 1.664 mmol) was added and the brownish solution was shaken for 36 h at rt. The product anthraquinone precipitated from the reaction mixture and was isolated by filtration and washing with CH_2Cl_2 (2 mL). The filtrate also contained the product. The solvent was removed from the filtrate and the residue chromatographed on silica using CH_2Cl_2 as eluent. The combined yield was 0.063 g (0.303 mmol, 54%) [1].

NMR (δ, $CDCl_3$) 8.35-8.32 (m, 4H, aromatic), 7.83-7.80 (m, 4H, aromatic); $^{13}C\{^1H\}$ 183.4 (s, *C*O), 134.4 (s, aromatic), 133.8 (s, aromatic), 127.5 (s, aromatic) ; IR (cm^{-1}, neat liquid) $\nu_{C\equiv O}$ 1673 (m), 1574 (m, sh).

The substrate cinnamyl alcohol (0.100 g, 0.746 mmol) and the catalyst **4a** (0.009 g, 0.015 mmol) were dissolved in pyridine-d_5 (1.0 mL). The oxidant *t*-BuOOH (0.41 mL of 5.5M solution in decane, 2.24 mmol) was added and the brownish solution was shaken for 42 h at rt and a ^{1}H-NMR of the reaction mixture was recorded. The NMR yield was determined by comparing the peak intensity of the C*H*$_2$OH group of cinnamyl alcohol with the peak intensity of the –C*H*O group of cinnamaldehyde (31%). The ^{1}H NMR spectrum is given below.

The substrate phenylmethanol (0.100 g, 0.925 mmol) and the catalyst **4a** (0.011 g, 0.019 mmol) were dissolved in pyridine-d_5 (1.0 mL). The oxidant *t*-BuOOH (0.50 mL of 5.5M solution in decane, 2.8 mmol) was added and the brownish solution was shaken for 42 h at rt and a ^{1}H-NMR of the reaction mixture was recorded. The NMR yield was determined by comparing peak intensity of the C*H*$_2$OH group of phenylmethanol with the peak intensity of the –C*H*O group of benzaldehyde (47%). The ^{1}H NMR spectrum is given below.

X-Ray determination for Complex ***4a***

X-ray quality crystals of **4a** were obtained by layering a CH_2Cl_2 solution with hexanes, which was stored at –18 °C for one week.

Preliminary examination and X-ray data collection were performed using a Bruker Kappa Apex II single crystal X-Ray diffractometer equipped with an Oxford Cryostream LT device. Intensity data were collected by a combinations of ϖ and ϕ scans. Apex II, SAINT and SADABS software packages were used for data collection, integration and correction of systematic errors, respectively [2].

Crystal data and intensity data collection parameters are listed in Table S1. Structure solution and refinement were carried out using the SHELXTL- PLUS software package [3]. The structure was solved by direct methods and refined successfully in the space group $P2_12_12_1$. Due to weak diffraction and poor quality data only the heavy atoms (Fe, Br, P, Cl) were refined with anisotropic displacement parameters. All other non-hydrogen atoms were refined with isotropic displacement parameters. The hydrogen atoms were treated using appropriate riding model (AFIX m3).

Complete listings of positional and isotropic displacement coefficients for hydrogen atoms, anisotropic displacement coefficients for the non-hydrogen atoms and tables of calculated and observed

structure factors are available in electronic format.CCDC 727535 contains the supplementary crystallographic data for **2a**. The data can be obtained free of charge from The Cambridge Crystallographic Data Centre via www.ccdc.cam.ac.uk/data_request/cif.

Table S1. Crystal data and structure refinement for **4a**

Empirical formula	$C_{31}H_{29}BrCl_6FeNO_3P$
Formula weight	842.98
Temperature, Wavelength	100(2) K, 0.71073 Å
Crystal system, Space group	Orthorhombic, $P2_12_12_1$
Unit cell dimensions	a = 9.2801(12) Å
	b = 11.0324(15) Å
	c = 32.916(4) Å
Volume, Z	3370.0(8) Å^3, 4
Density (calculated)	1.661 Mg/m^3
Absorption coefficient	2.191 mm^{-1}
Crystal size	0.26 × 0.08 × 0.06 mm^3
Theta range for data collection	1.24 to 25.00°.
Reflections collected	64463
Independent reflections	5921 [R(int) = 0.2193]
Absorption correction	Semi-empirical from equivalents
Max. and min. transmission	0.8743 and 0.5965
Data / restraints / parameters	5921 / 6 / 209
Goodness-of-fit on F2	1.556
Final R indices [I>2sigma(I)]	R1 = 0.1517, wR2 = 0.3669
R indices (all data)	R1 = 0.2221, wR2 = 0.3961
Absolute structure parameter	0.14(4)
Largest diff. peak and hole	1.949 and -3.464 e.Å^{-3}

Figure S1. Molecular structure of one of the diastereomers of **4a** (depicted with 30% probability ellipsoids, H atoms, and solvents are omitted for clarity). Key bond lengths (Å) and bond angles (deg): Br1-Fe1, 2.447(3); Fe1-C1, 1.72(2); O1-C1, 1.17(3); Fe1-P1, 2.148(6); P1-N1, 1.578(17); C1-Fe1-P1, 92.8(7); C1-Fe1-Br1, 88.4(7); P1-Fe1-Br1, 92.68(17); O1-C1-Fe1, 176(2); N1-P1-Fe1, 118.7(6); O3-P1-O2, 100.5(6).

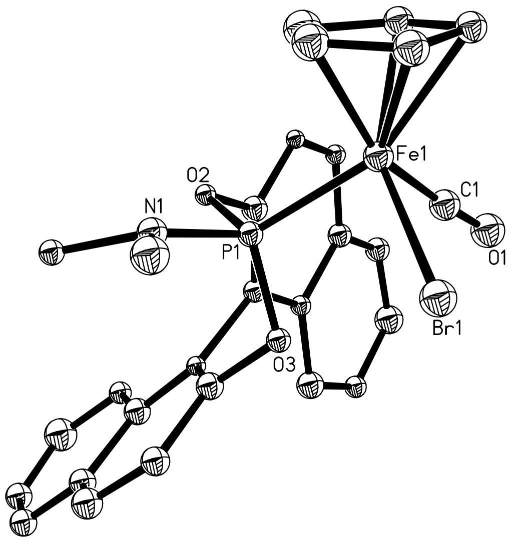

"**[FeBr(Cp)(CO)(1a)]**" **(4a)** ^{1}H and ^{13}C NMR

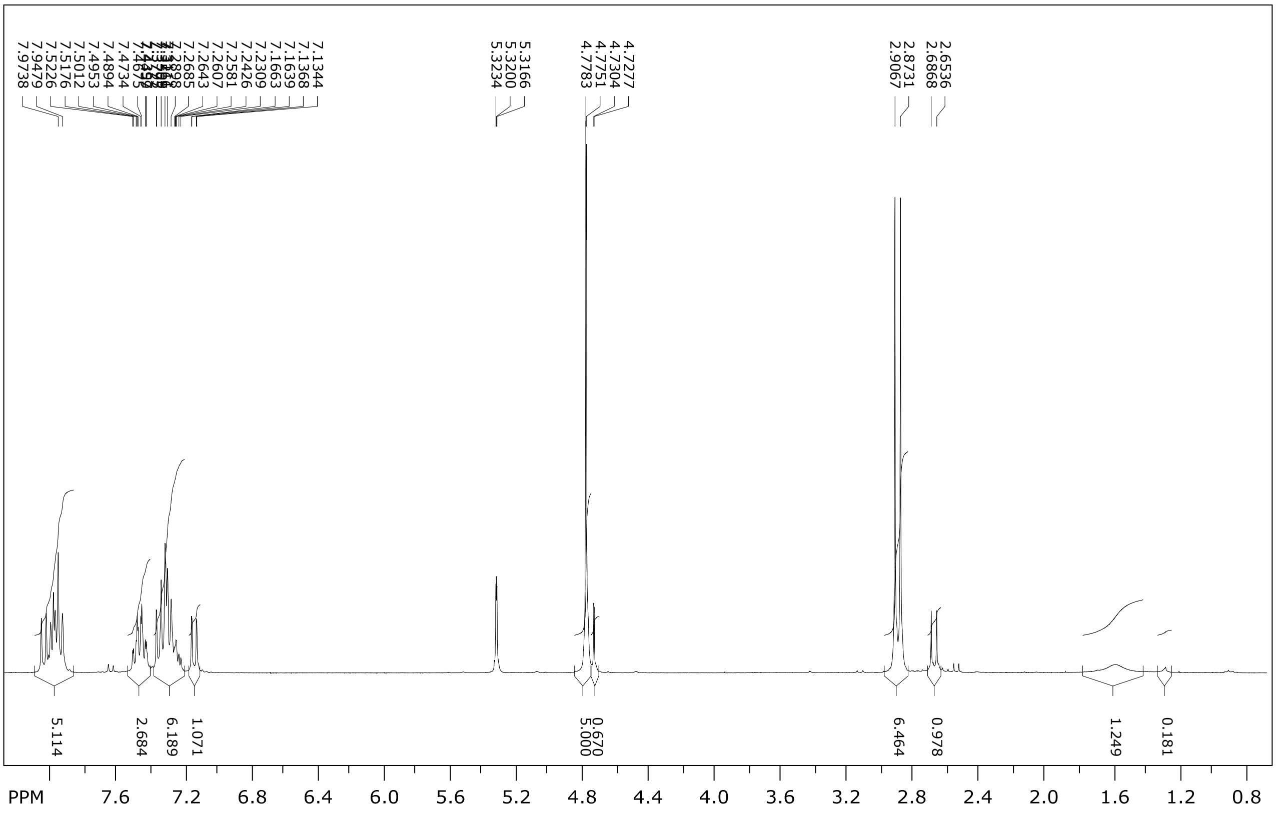

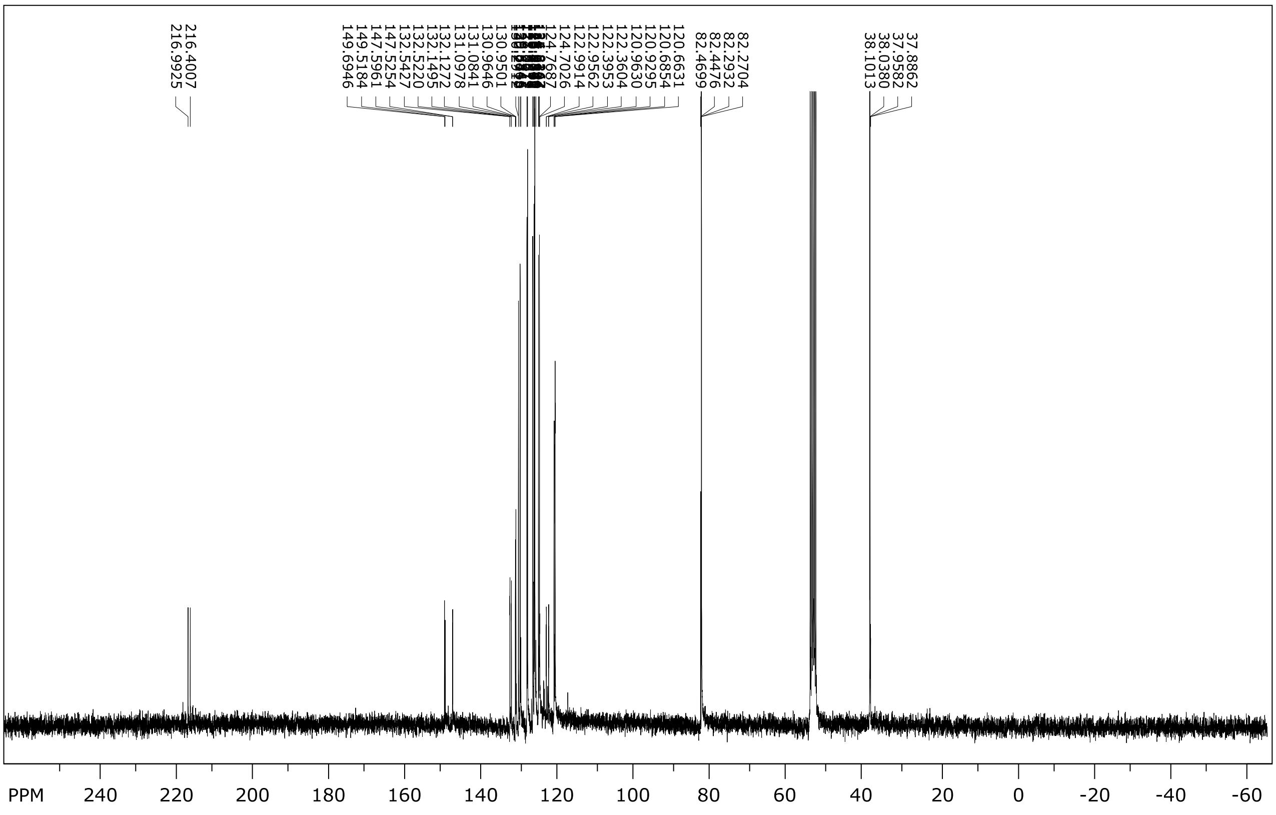

"**[FeBr(Cp)(CO)(1b)]**" (**4b**) ^{1}H and ^{13}C NMR

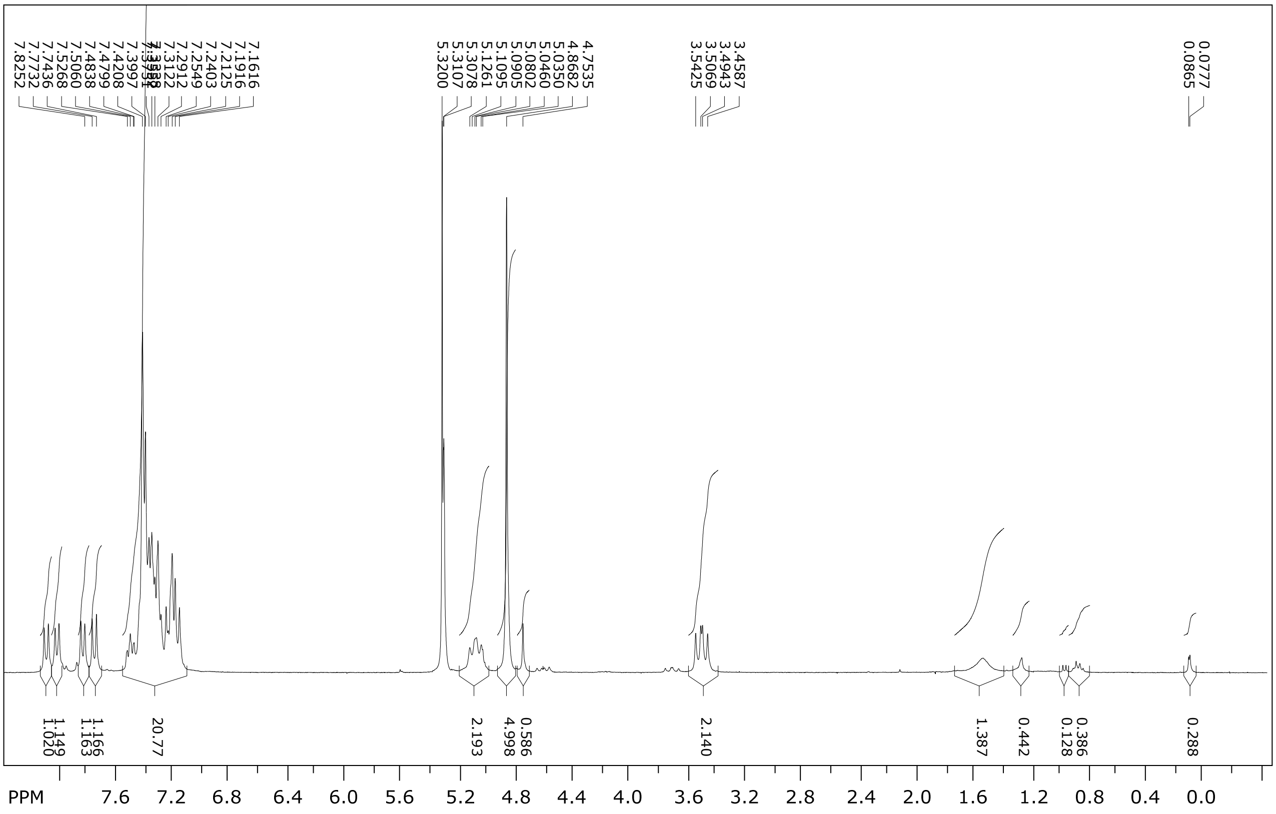

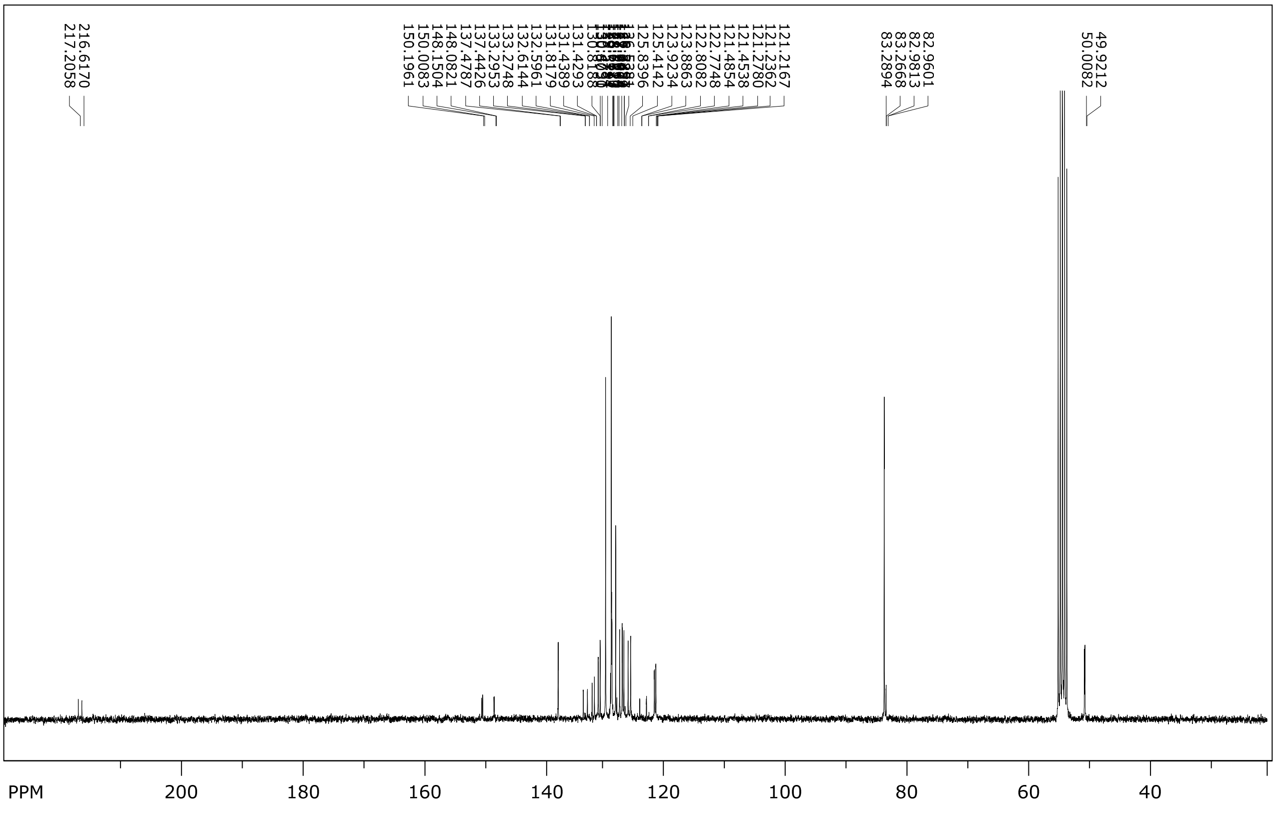

"**[Fe(Cp)I(CO)(1b)]**" **(5b)** ^{1}H and ^{13}C NMR

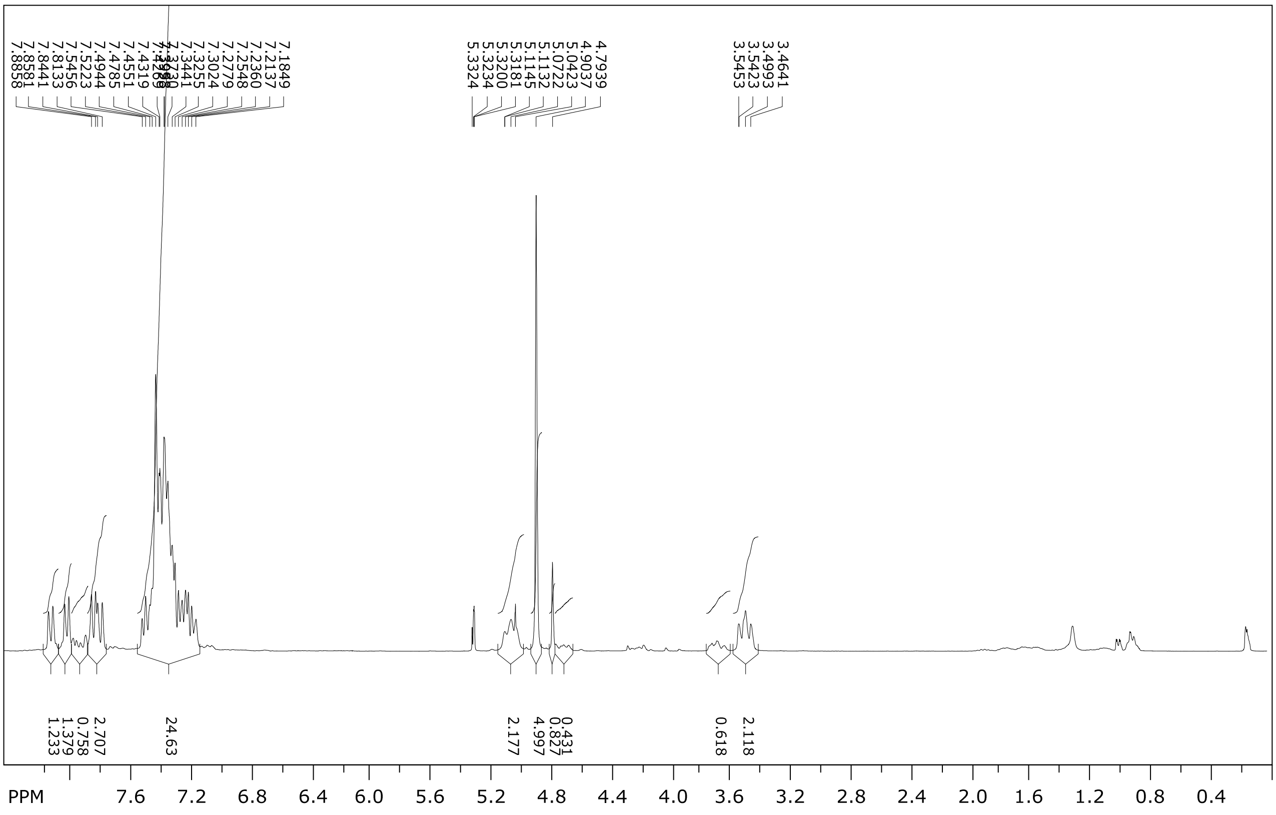

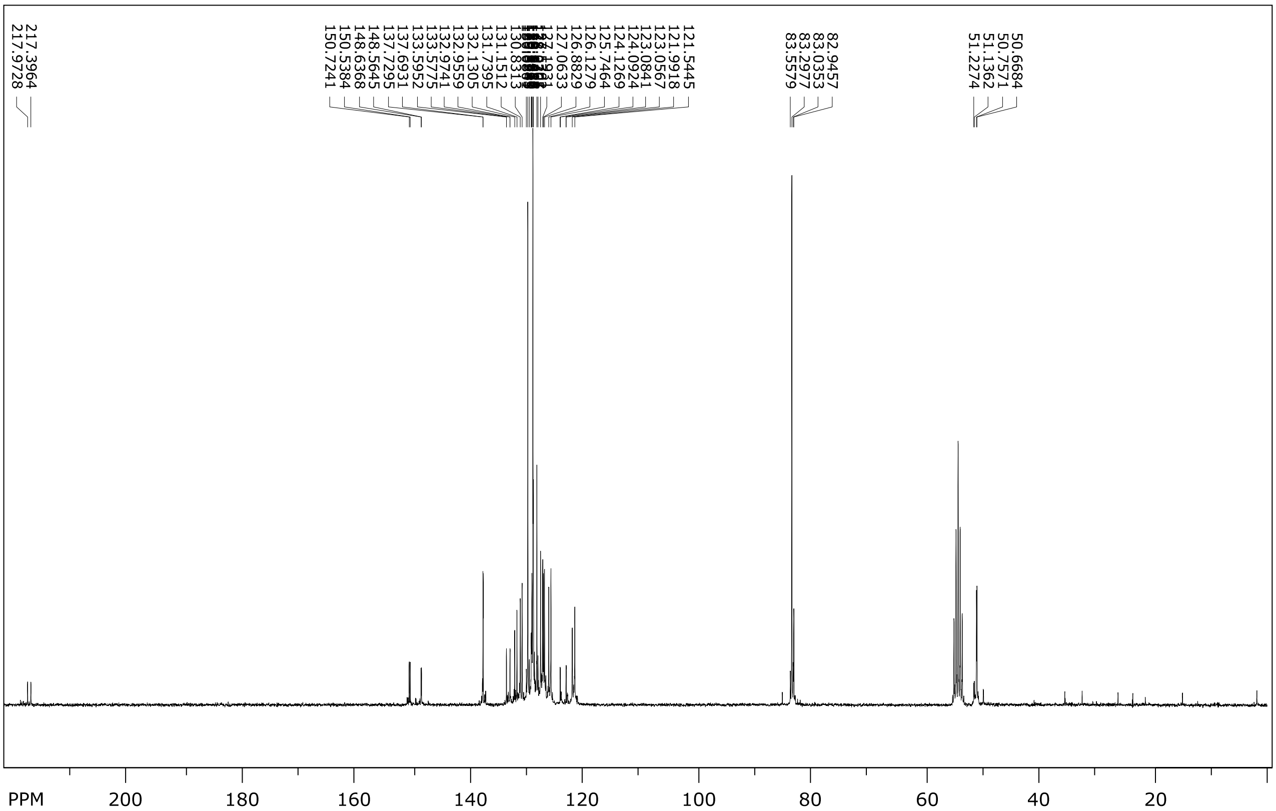

Benzophenone ^{1}H and ^{13}C NMR (Table 4, entry 1, in acetone-d_6)

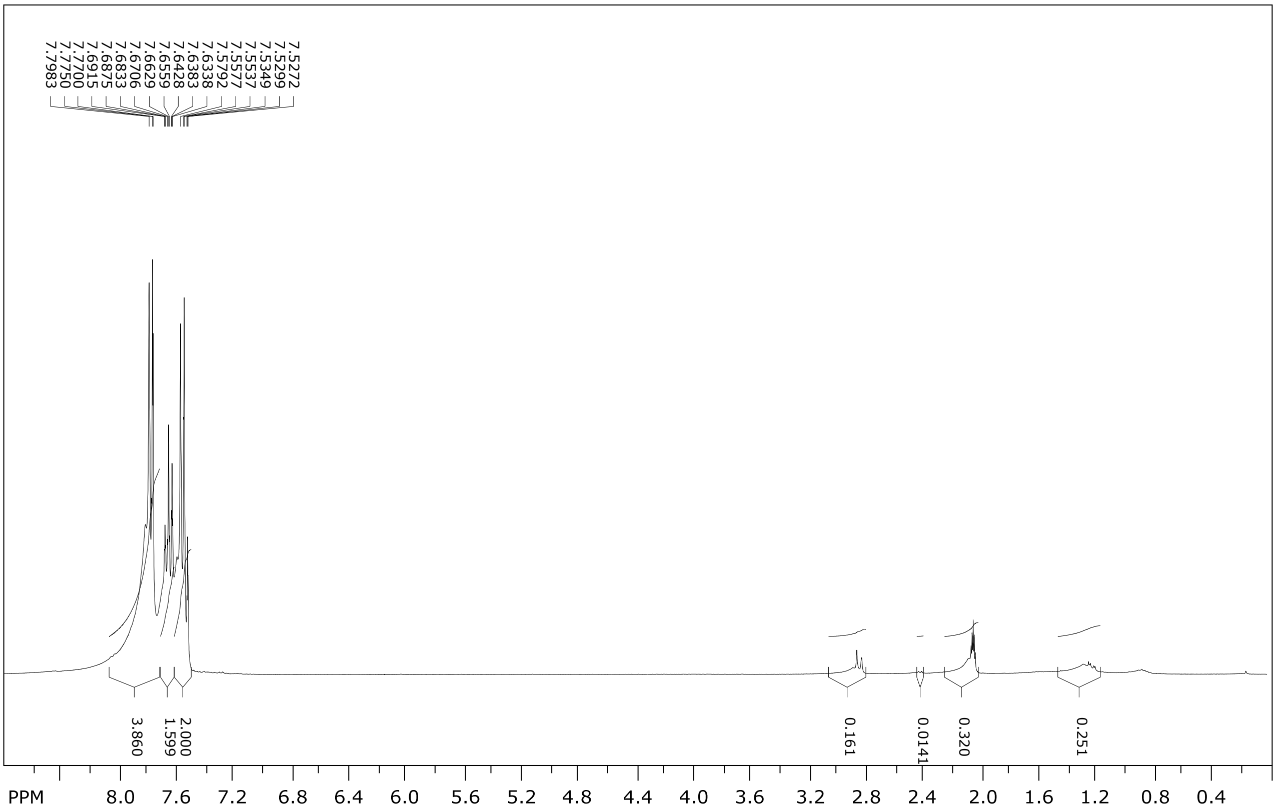

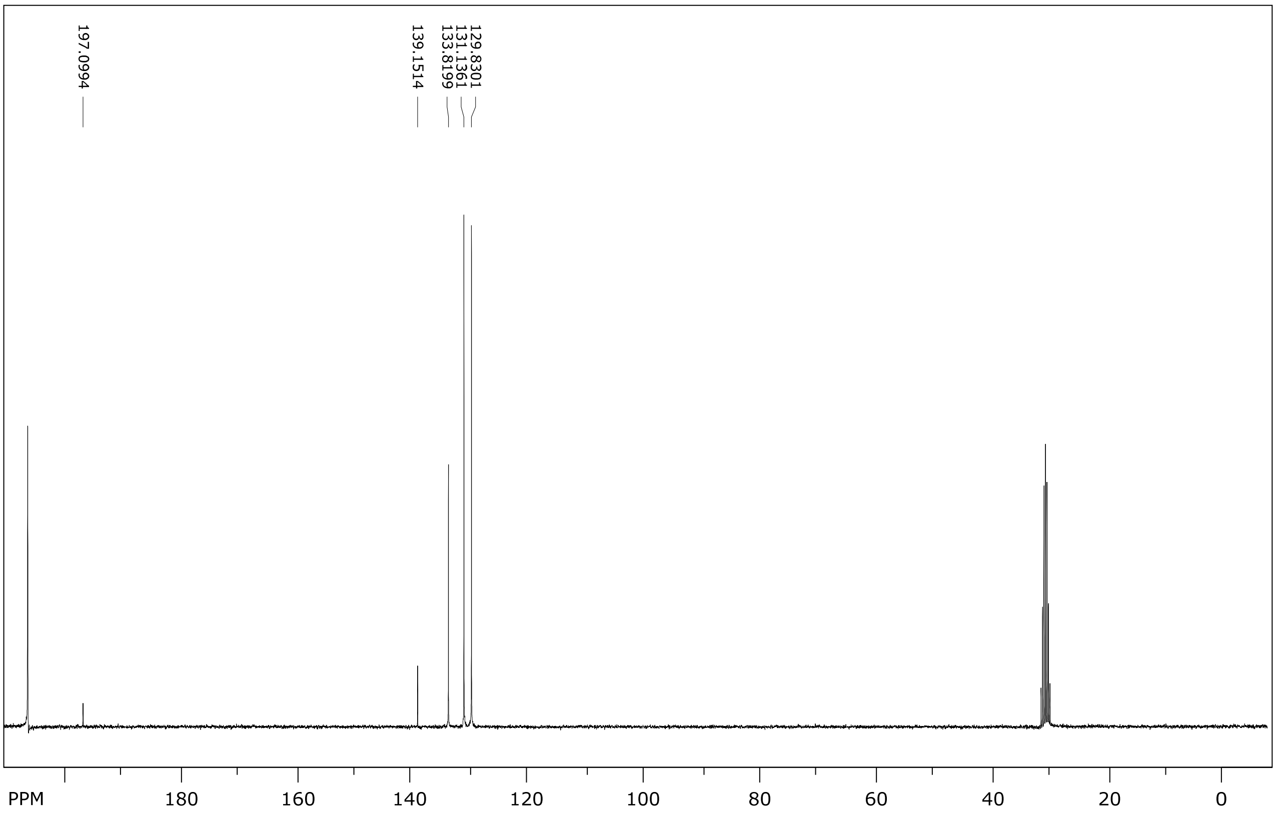

Fluorenone ^{1}H and ^{13}C NMR (Table 4, entry 2)

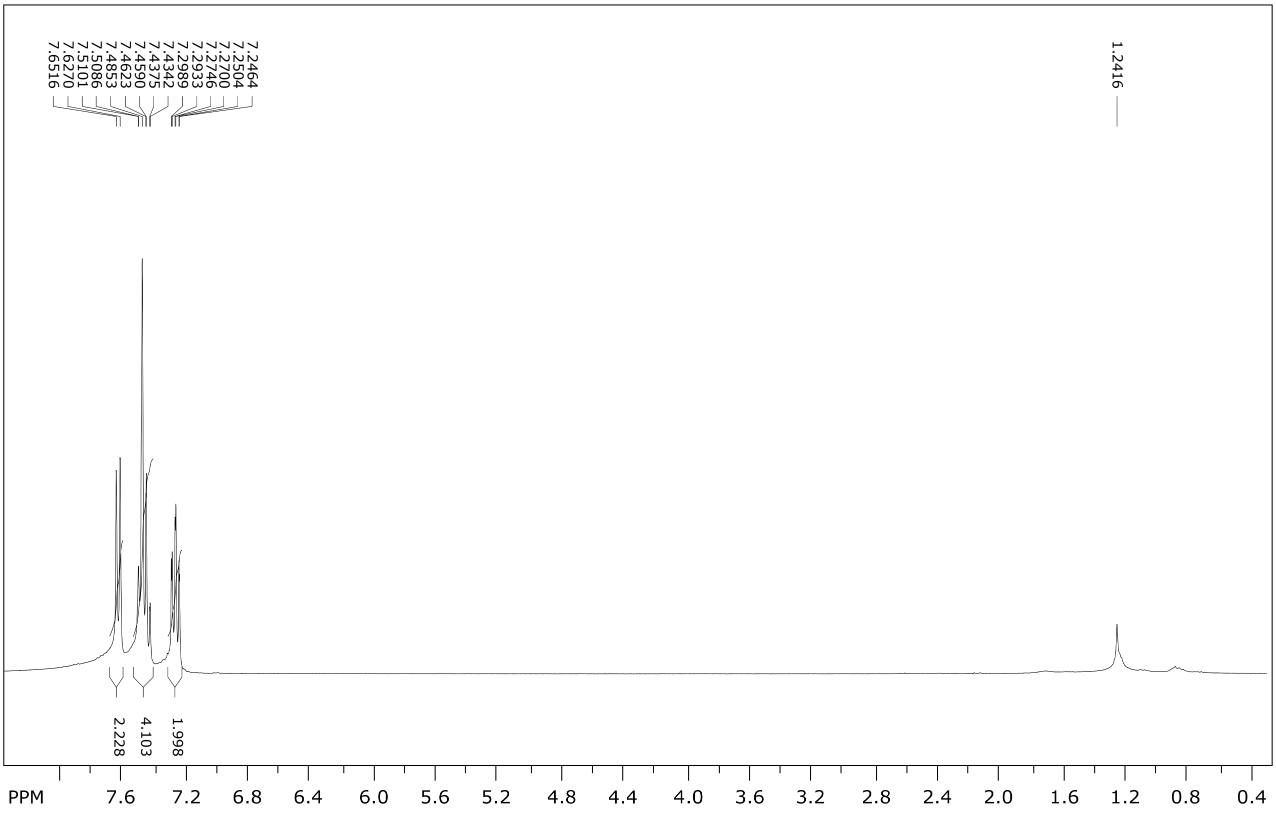

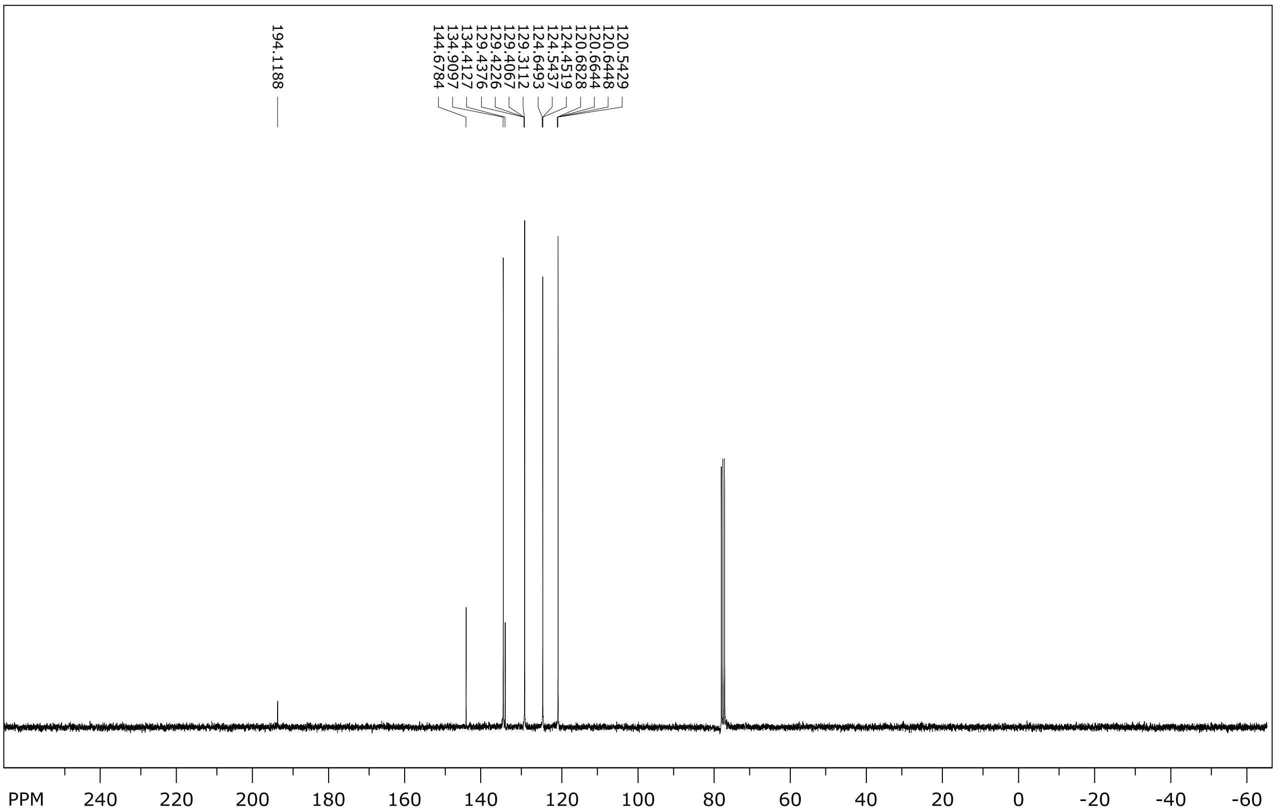

Anthraquinone ^{1}H and ^{13}C NMR (Table 4, entry 3)

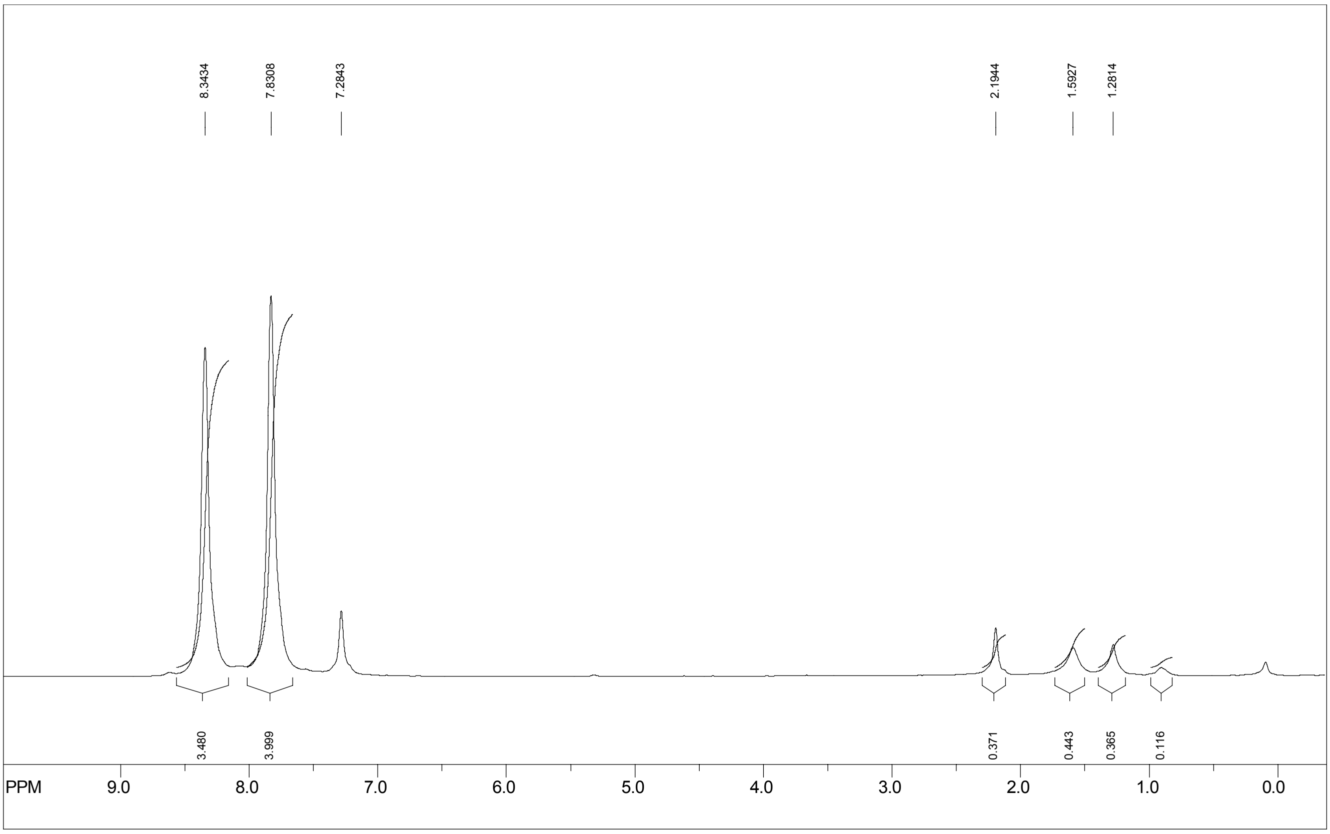

file: C:\Documents and Settings\pssgk8\Desktop\PushkarNMR\Catalytic activity products\Anthraquinone\PSS-LJ-08-081H1Antrhaquinone.fid\fid [illegible]
transmitter freq.: 299.945913 MHz
time domain size: 29952 points
width: 4000.00 Hz = 13.335738 ppm = 0.133547 Hz/pt
number of scans: 16

processed size: 32768 complex points
LB: 10.000 GB: 0.0000

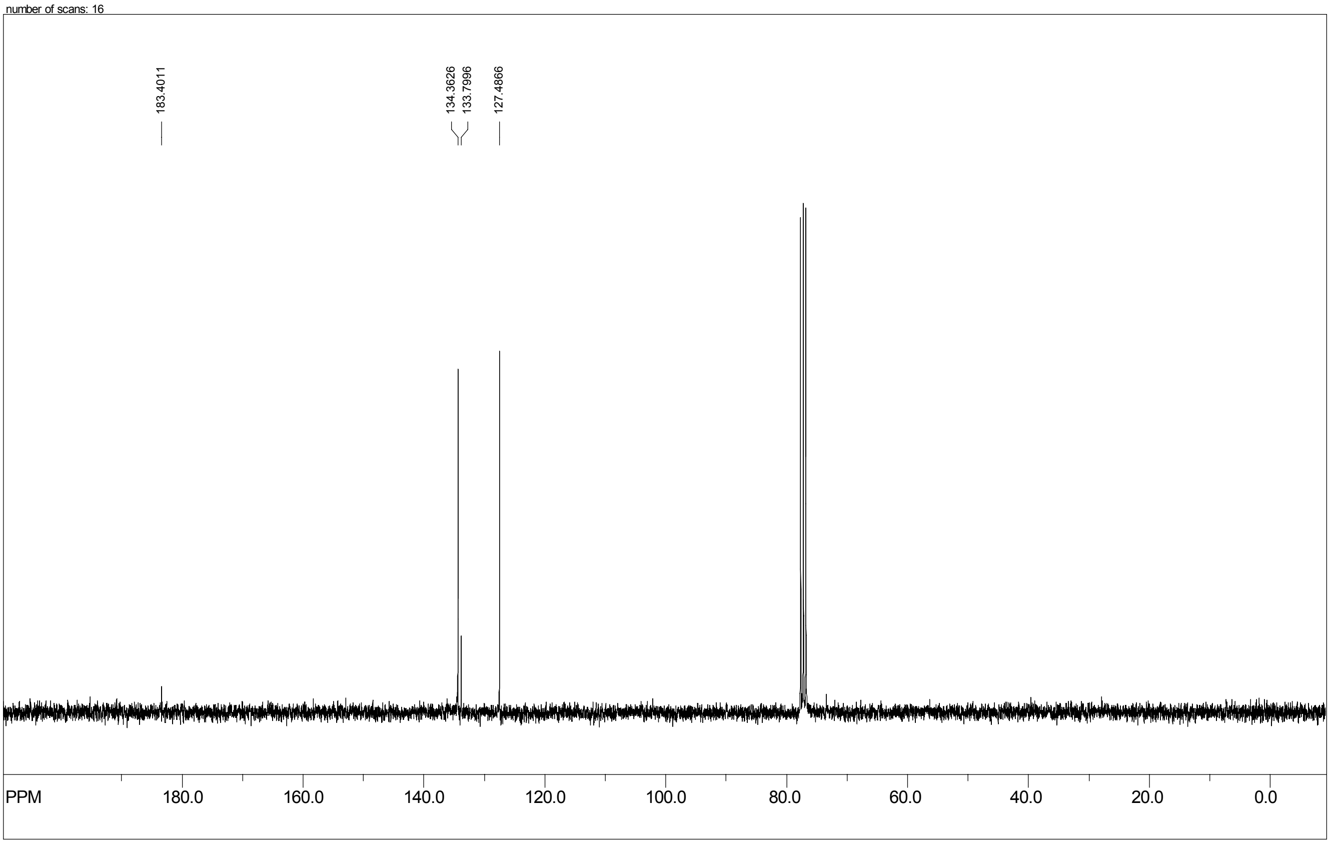

file: C:\Documents and Settings\pssgk8\Desktop\PushkarNMR\Catalytic activity products\Anthraquinone\PSS-LJ-08-081anthraquinoneC13.fid\fid [illegible]
transmitter freq.: 75.428624 MHz
time domain size: 59900 points
width: 16501.65 Hz = 218.771724 ppm = 0.275487 Hz/pt
number of scans: 512

processed size: 65536 complex points
LB: 1.000 GB: 0.0000

Oxidation of cinnamyl alcohol in pyridine-d_5 (Table 4, entry 4).

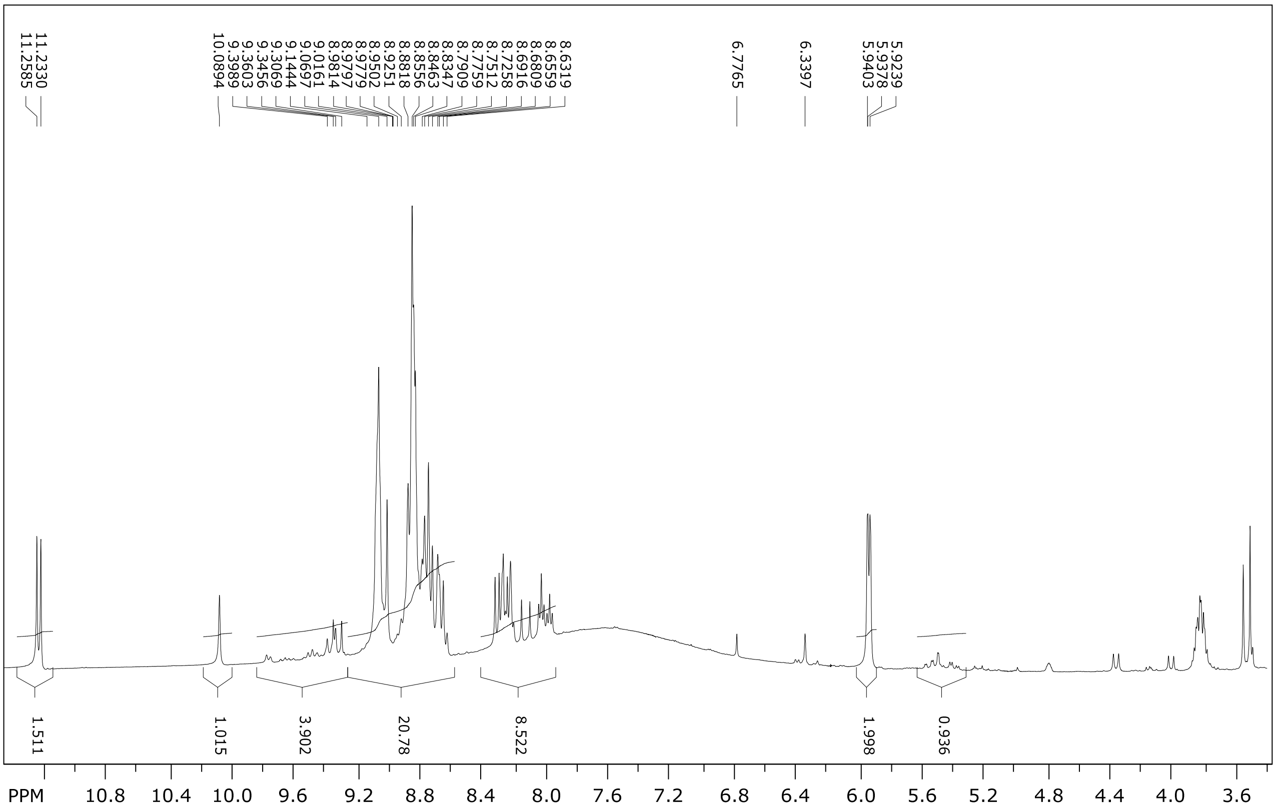

Oxidation of phenylmethanol in pyridine-d_5 (Table 3, entry 5)

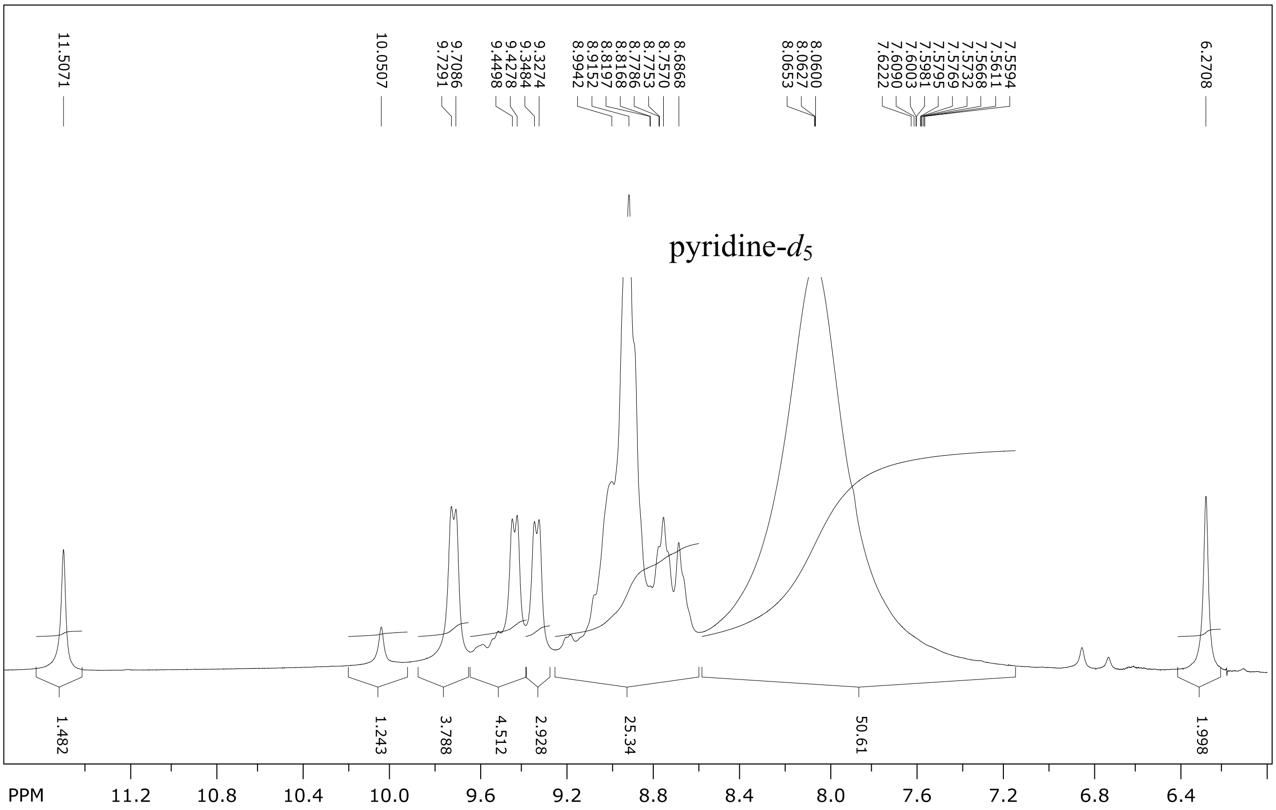

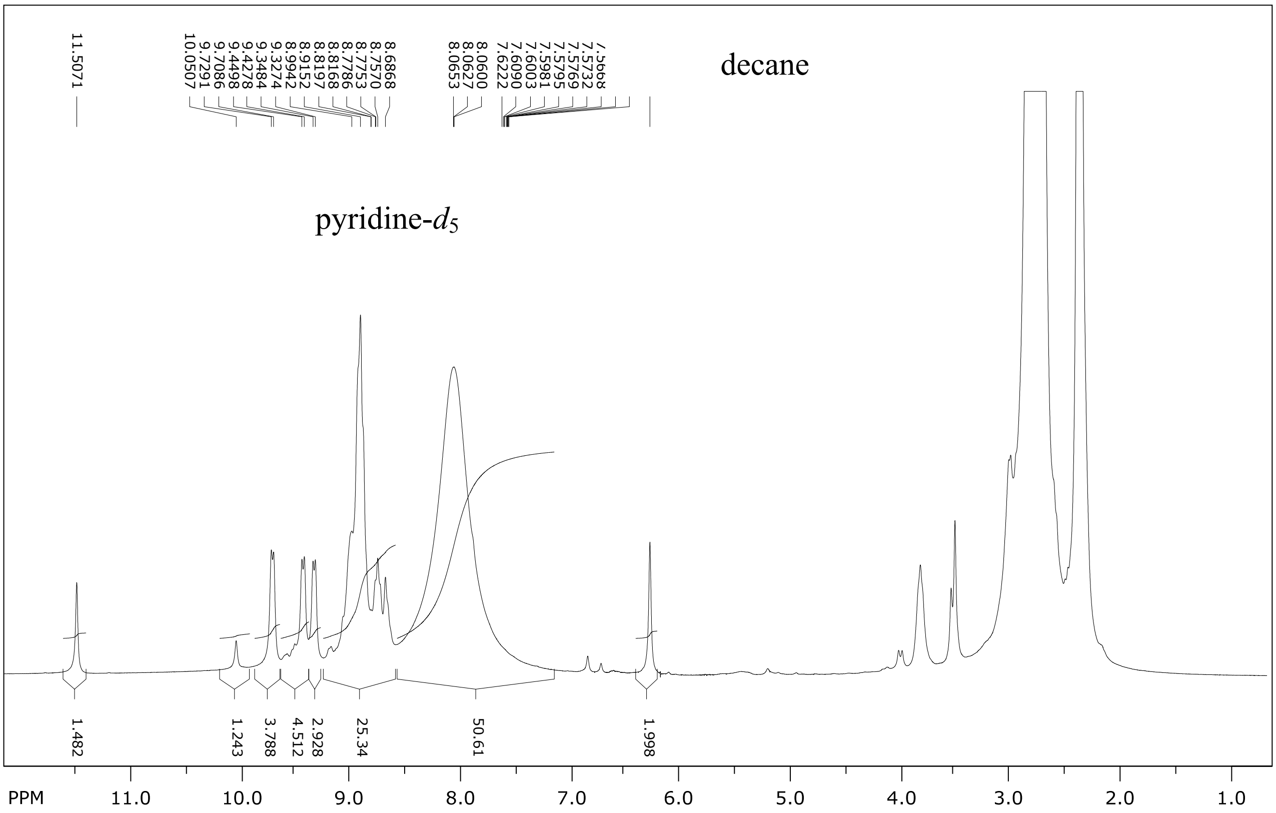

References

[1] The product contains about 10% anthracene (assessed by ^{1}H NMR).

[2] Bruker Analytical X-Ray, Madison, WI, 2008.

[3] G. M. Sheldrick, *Acta Cryst*. 2008, **A64**, 112.
